# Ligustrazine Combined with Sinomenine Alleviates Central Sensitization in CCI-Induced Neuropathic Pain in Rats by Restoring Metabolic Homeostasis in the Spinal Dorsal Horn

**DOI:** 10.3390/metabo16100717

**Published:** 2026-09-28

**Authors:** Zhaoyue Yuan, Chang Gao, Xiaoliang Zhao, Jingyi Wang, Yue Jiao, Yang Liu, Jingzhe Li, Runzi Bai, Zhiguo Wang, Tao Li

**Affiliations:** 1 Experimental Research Center, China Academy of Chinese Medical Sciences, Beijing 100700, China; 2 Institute of Chinese Materia Medica, China Academy of Chinese Medical Sciences, Beijing 100700, China

**Keywords:** neuropathic pain, central sensitization, metabolic remodeling

## Abstract

Background: Neuropathic pain (NP) is a chronic pain condition caused by a lesion or disease of the somatosensory nervous system. Its development and persistence are closely associated with peripheral nociceptive input, central sensitization, neuroimmune dysregulation, and remodeling of the local metabolic microenvironment. Our previous studies demonstrated that combined treatment with ligustrazine and sinomenine ameliorates early sciatic nerve pathological damage and systemic metabolic disturbances in rats with chronic constriction injury (CCI). However, whether this combination modulates central sensitization in NP remains unclear. Objective: This study aimed to evaluate the effects of combined ligustrazine and sinomenine treatment on pain-related behaviors and motor function during the maintenance phase of CCI-induced neuropathic pain and to investigate the potential mechanisms underlying its attenuation of central sensitization, with particular emphasis on metabolic remodeling, neurotransmitter homeostasis, and associated regulatory molecules in the spinal dorsal horn. Methods: A rat model of CCI was established, and animals were treated with ligustrazine combined with sinomenine (12.5 + 12.5, 25 + 25, or 50 + 50 mg/kg) for 7 consecutive days. Mechanical withdrawal threshold, cold hypersensitivity, and gait behavior were assessed. A multiplex assay was used to profile 12 plasma inflammatory cytokines and pain-related mediators. Integrated transcriptomic and metabolomic analyses of the spinal dorsal horn were subsequently performed to identify key altered pathways. Oxylipin profiling, targeted neurotransmitter analysis, and quantification of metabolites associated with the arginine–polyamine pathway were further conducted to characterize alterations in the local metabolic microenvironment associated with central sensitization. Finally, quantitative real-time PCR (qRT-PCR) and Western blotting were used to evaluate molecules related to polyamine metabolism, redox homeostasis, and central sensitization. Results: Combined ligustrazine and sinomenine treatment improved pain-related behaviors and gait dysfunction in CCI rats and partially normalized systemic inflammatory dysregulation. The ipsilateral-to-contralateral maximum contact-area ratio decreased from 102.8 ± 9.0% in the Sham group to 53.1 ± 9.7% in the Model group and recovered to 85.5 ± 4.4% in the high-dose group. Metabolomic and transcriptomic analyses identified 54 differential metabolites and 130 differentially expressed genes, respectively, following treatment. Integrated multi-omics analysis highlighted arginine-related metabolic pathways, together with alterations in neurotransmitter, polyamine, oxylipin, and redox homeostasis.

## 1. Introduction

Neuropathic pain (NP) is a chronic pain condition caused by a lesion or disease of the somatosensory nervous system and is commonly characterized by spontaneous pain, hyperalgesia, and allodynia, substantially impairing patients’ quality of life and social functioning [1]. Unlike nociceptive pain, the development and persistence of NP are not only associated with abnormal nociceptive input following peripheral nerve injury but also involve sustained structural and functional alterations within the nervous system. Following peripheral nerve injury, inflammatory responses, immune-cell activation, and increased excitability of sensory neurons occur in the injured nerve and its surrounding microenvironment, thereby contributing to peripheral sensitization. Persistent abnormal nociceptive input subsequently acts on the spinal dorsal horn and associated central pain-processing pathways, inducing neuronal hyperexcitability, glial activation, aberrant synaptic transmission, and dysregulated neuroimmune interactions, which collectively contribute to the development and maintenance of central sensitization [2,3]. Long-lasting synaptic and circuit remodeling further contributes to the persistence of chronic pain [4]. In addition to inflammation and altered neuronal excitability, disruption of neurotransmitter homeostasis, enhanced oxidative stress, and remodeling of the local metabolic microenvironment are also closely associated with the pathogenesis of NP [5,6,7]. Thus, NP represents a complex chronic pain state driven by both persistent peripheral injury-related input and aberrant central regulation.

Arginine metabolism and redox imbalance may represent important mechanistic links among neuroinflammation, metabolic remodeling, and central sensitization. Arginine is not only involved in nitric oxide synthesis and the urea cycle but can also be metabolized through the ornithine pathway to generate polyamines, including putrescine, spermidine, and spermine. Polyamines participate in the regulation of ion channels, NMDA receptor function, inflammatory responses, and oxidative stress, and disturbances in polyamine metabolism may therefore influence neuronal excitability and synaptic plasticity in the spinal dorsal horn [8]. Meanwhile, peripheral nerve injury can increase reactive oxygen species production, impair reducing capacity, and disrupt glutathione cycling, thereby promoting glial activation, inflammatory mediator release, and neuronal hyperexcitability [6]. In particular, SLC7A11/xCT, the key light-chain subunit of system xc^−^, mediates cystine uptake coupled with glutamate export, thereby providing substrate for glutathione synthesis while potentially influencing extracellular glutamate levels and excitatory neurotransmission [3,5,9]. These observations suggest that arginine–polyamine metabolism, glutathione-associated redox processes, and glutamate homeostasis may constitute an interconnected metabolic regulatory network that contributes to abnormalities in the local spinal dorsal horn microenvironment and to the maintenance of central sensitization in NP.

In East Asian traditional medicine, Rhizoma Chuanxiong and Sinomenii Caulis have long been used clinically for pain management. Ligustrazine is one of the major active constituents of Rhizoma Chuanxiong, and its clinically used preparations, such as ligustrazine injection [10], exhibit multiple pharmacological activities, including anti-inflammatory, antioxidant, anti-apoptotic, microcirculation-improving, and neuroprotective effects. Sinomenine is a representative alkaloid derived from Sinomenii Caulis, and its clinical preparation, Zhengqing Fengtongning [11], possesses anti-inflammatory, immunomodulatory, analgesic, and neuroprotective activities. Unlike conventional single-target agents, both ligustrazine and sinomenine exhibit multi-pathway regulatory properties [12], providing a theoretical rationale for their combined use in the treatment of NP. Our previous studies demonstrated that combined treatment with ligustrazine and sinomenine exerted marked antinociceptive effects in multiple models of peripheral and central NP [13]. More recently, we showed that this combination ameliorated mechanical hypersensitivity and cold hypersensitivity in CCI rats, attenuated sciatic nerve pathological damage and inflammatory responses, and, based on integrated network pharmacology and metabolomic analyses, may exert its effects through modulation of inflammation, amino acid metabolism, and neurotransmitter-related pathways [14]. However, the persistence of NP depends not only on peripheral inflammation and nerve injury but also on central sensitization and remodeling of the local metabolic microenvironment within the spinal dorsal horn. Whether combined ligustrazine and sinomenine treatment can further restore metabolic homeostasis in the spinal dorsal horn and modulate central mechanisms associated with NP therefore remains to be elucidated.

Accordingly, the present study employed a rat model of CCI to evaluate the effects of 7 consecutive days of combined ligustrazine and sinomenine treatment on pain-related behaviors, gait function, and systemic inflammatory responses. Focusing on the spinal dorsal horn as a critical site of central sensitization, we further integrated transcriptomic and metabolomic analyses with oxylipin profiling, targeted neurotransmitter analysis, assessment of arginine–polyamine metabolism, quantitative real-time PCR (qRT-PCR), and Western blotting to systematically investigate the potential central mechanisms underlying the therapeutic effects of this combination. This study aimed to elucidate the mechanistic basis of combined ligustrazine and sinomenine treatment in NP from the perspective of metabolic microenvironment remodeling in the spinal dorsal horn, thereby providing experimental evidence for its further development and clinical application.

## 2. Materials and Methods

### 2.1. Animals and Treatment

Specific pathogen-free (SPF) male Sprague–Dawley rats, aged 6–7 weeks and weighing 180–220 g, were purchased from Beijing HFK Bioscience Co., Ltd. (Beijing, China; animal production license No. SCXK [Beijing] 2024-0003). Rats were housed three per cage under SPF conditions with free access to food and water and maintained on a 12-h light/12-h dark cycle. Before CCI or sham surgery, all rats were allowed to acclimatize to the animal facility for 3 days under the above housing conditions. All animal experimental procedures were approved by the Animal Ethics Committee of the Experimental Research Center, China Academy of Chinese Medical Sciences (No. ERCCACMS21-2111-01).

Following successful establishment of the CCI model, 40 rats were randomly assigned to five groups (*n* = 8 per group): the model group (Model, normal saline, 10 mL·kg^−1^·d^−1^), the low-dose ligustrazine plus sinomenine group (LGZ + SIN; LGZ 25 mg·kg^−1^·d^−1^ + SIN 25 mg·kg^−1^·d^−1^), the medium-dose LGZ + SIN group (LGZ 50 mg·kg^−1^·d^−1^ + SIN 50 mg·kg^−1^·d^−1^), the high-dose LGZ + SIN group (LGZ 100 mg·kg^−1^·d^−1^ + SIN 100 mg·kg^−1^·d^−1^), and the positive-control pregabalin group (Pgb, 30 mg·kg^−1^·d^−1^). An additional eight healthy rats were assigned to the sham-operated group (Sham, normal saline, 10 mL·kg^−1^·d^−1^). All treatments were administered by oral gavage twice daily, once in the morning and once in the evening, for 7 consecutive days.

### 2.2. Reagents and Materials

Ligustrazine hydrochloride (batch No. DT201803-19) and sinomenine hydrochloride (batch No. DT201806-22), each with a purity of ≥98%, were purchased from Shaanxi Datian Biotechnology Co., Ltd. (Xi’an, China). Pregabalin (batch No. 295422) was obtained from J&K Scientific Ltd. (Beijing, China). Isoflurane (batch No. 217180801) was purchased from RWD Life Science Co., Ltd. (Shenzhen, China). Chromic catgut sutures (4-0) were obtained from Boda Medical Products Co., Ltd. (Shandong, China). The RayPlex™ multiplex bead-based assay used for plasma multi-analyte detection was purchased from RayBiotech (Peachtree Corners, GA, USA). For transcriptomic analysis of the spinal dorsal horn, QIAzol Lysis Reagent was obtained from QIAGEN GmbH (Hilden, Germany), the RNA Purification Kit from Majorbio Bio-Pharm Technology Co., Ltd. (Shanghai, China), the SMART-Seq™ v4 Ultra Low Input RNA Kit for Sequencing from Clontech (Mountain View, CA, USA), the DNBSEQ Reagent Kit from MGI Tech Co., Ltd. (Shenzhen, China), and agarose from Biowest (Nuaillé, France).

Methanol, acetonitrile, formic acid, ammonium formate, isopropanol, ethyl acetate, and other solvents used for metabolomics, oxylipin profiling, targeted neurotransmitter analysis, and arginine–polyamine metabolism analysis were of chromatographic or LC–MS grade. Standards and internal standards for polyunsaturated fatty acids and their oxygenated metabolites were purchased from Cayman Chemical (Ann Arbor, MI, USA), including AA-d8, 20-HETE-d6, leukotriene B4-d4, and PGE2-d5. Standards for neurotransmitter-related metabolites, including tyrosine and tryptophan, as well as sodium tetraborate, benzoyl chloride, and d5-benzoyl chloride, were obtained from Sigma-Aldrich (St. Louis, MO, USA). All analytical standards and internal standards had purities > 98%. Ultrapure water was generated using a Milli-Q water purification system.

A bicinchoninic acid (BCA) protein assay kit was purchased from Thermo Fisher Scientific (Waltham, MA, USA). The cDNA reverse-transcription kit and SYBR Green real-time PCR master mix used for qRT-PCR were obtained from TOYOBO (Osaka, Japan). Primers for the rat reference gene ACTB and target genes were synthesized by Sangon Biotech Co., Ltd. (Shanghai, China). RIPA lysis buffer and ECL Plus chemiluminescent substrate used for Western blotting were purchased from Solarbio Science & Technology Co., Ltd. (Beijing, China), and the one-step rapid Western blotting HRP kit was obtained from CWBIO (Beijing, China). The SAT1 polyclonal antibody was purchased from Proteintech (Rosemont, IL, USA), the xCT antibody from Abcam (Cambridge, UK), and the recombinant rabbit monoclonal NR2B antibody from HuaAn Biotechnology Co., Ltd. (Hangzhou, China).

The major instruments and equipment used in this study included an EasyFlow individually ventilated cage system from Tecniplast (Buguggiate, Italy); von Frey filaments from Ugo Basile Biological Apparatus (Gemonio, Italy); a tabletop anesthesia machine from Harvard Apparatus (Holliston, USA); a ThermoStar body temperature maintenance system from RWD Life Science Co., Ltd. (Shenzhen, China); a rodent gait analysis system from Beijing Zhongshi Dichuang Technology Development Co., Ltd. (Beijing, China); a custom-built pain testing platform; a Targin VX-III multi-tube vortex mixer from Targin Technology Co., Ltd. (Beijing, China); a Rotanta 460R refrigerated high-speed centrifuge from Hettich (Tuttlingen,, Germany); an MC-8 integrated refrigerated centrifugal concentrator from Beijing JM Technology Co., Ltd. (Beijing, China); a Synergy 2 multimode microplate reader from BioTek(Winooski, VT, USA); and a high-performance liquid chromatography–tandem mass spectrometry (HPLC–MS/MS) system from AB Sciex (Framingham, MA, USA), comprising an ExionLC-20AC high-performance liquid chromatograph, an Ion Drive™ Turbo V ion source, a SCIEX 6500+ triple-quadrupole mass spectrometer, Analyst 1.7 data acquisition software, and MultiQuant 3.0.3 data processing software all from AB Sciex (Framingham, MA, USA).

Additional instruments included a BD FACSymphony™ A1 flow cytometer from BD Biosciences (San Jose, CA, USA); a DYY-6C electrophoresis power supply from Liuyi Biotechnology Co., Ltd. (Beijing, China); a NanoDrop 2000 microvolume spectrophotometer from Thermo Fisher Scientific (Waltham, MA, USA); an Agilent 5300 Bioanalyzer from Agilent Technologies (Santa Clara, CA, USA); a Qubit 4.0 fluorometer from Thermo Fisher Scientific (Waltham, MA, USA); a T100 Thermal Cycler from Bio-Rad (Hercules, CA, USA); a QIAcube automated nucleic acid purification system from QIAGEN (Hilden, Germany); a NanoDrop One/OneC microvolume UV–Vis spectrophotometer from Thermo Fisher Scientific (Waltham, MA, USA); a Mastercycler nexus PCR system from Eppendorf (Hamburg, Germany); a CFX Connect Real-Time PCR Detection System from Bio-Rad (Hercules, CA, USA); Mini P-4 electrophoresis and wet-transfer units from Cavoy (Beijing, China); an electrophoresis power supply from Bio-Rad (Hercules, CA,, USA); a horizontal destaining shaker from Qilinbeier (Beijng, China); and an electric tissue homogenizer from Fluka (Buchs, Switzerland).

### 2.3. Establishment of the CCI Model

The rat model of chronic constriction injury (CCI) was established according to the method described by Bennett et al. [15]. Briefly, rats were anesthetized with inhaled isoflurane (5% for induction and 2.5% for maintenance) and placed on a heating pad to maintain body temperature at approximately 37 °C. A skin incision was made below the left femur, and the surrounding tissues were bluntly dissected to expose the left sciatic nerve. The nerve was loosely ligated at four sites using 4-0 chromic catgut sutures, with an interligature spacing of approximately 1 mm. The ligatures were tightened sufficiently to induce a slight twitch of the ipsilateral hindlimb muscles without compromising epineurial blood flow. In the Sham group, the sciatic nerve was exposed in the same manner but was not ligated.

Successful establishment of the CCI model was confirmed by comparing the mechanical withdrawal threshold (MWT) before surgery and on postoperative day 7. Rats exhibiting a reduction in MWT of ≥50% relative to the preoperative baseline were considered to have successfully developed the CCI model.

### 2.4. Pharmacodynamic Evaluation

#### 2.4.1. Assessment of Mechanical Withdrawal Threshold and Cold Hypersensitivity

The mechanical withdrawal threshold (MWT) was assessed using von Frey filaments [16]. Before testing, rats were individually placed in transparent plastic chambers (20 cm × 20 cm × 15 cm) with clear acrylic lids and allowed to acclimate for 30 min. Mechanical stimulation was then applied using von Frey filaments ranging from 4 to 26 g, and the MWT was calculated using the up-and-down method [17].

Cold hypersensitivity was evaluated using the acetone test. Briefly, 0.1 mL of acetone was applied to the plantar surface of the ipsilateral hind paw, and behavioral responses, including paw withdrawal and licking, were observed and scored according to response intensity. A score of 0 indicated no response; 1, a mild response or rapid withdrawal of the hind paw; 2, repeated flicking of the hind paw; and 3, sustained or repeated paw elevation accompanied by licking [18].

#### 2.4.2. Assessment of Gait Function

Gait analysis was performed using a rodent gait analysis system. Before testing, rats were allowed to acclimate to the testing environment. During each trial, the rat was placed at the starting end of an enclosed walkway and allowed to traverse the runway spontaneously. Footprint and gait information were recorded using a bottom-mounted imaging system and analyzed with the accompanying software (WalkAnalysator 1.0.9).

Each rat underwent three repeated trials. Valid recordings were defined as those showing stable locomotion without obvious stopping, turning, or jumping and containing 7–8 consecutive complete steps. The mean value from the three valid trials was used for subsequent analysis. The evaluated parameters included stance duration, swing duration, stance phase ratio, single-hind-paw support duration, and maximum contact area, which were used to assess gait coordination, hindlimb motor function, and weight-bearing capacity.

### 2.5. Plasma Multiplex Analysis

After completion of the treatment regimen and behavioral assessments, blood samples were collected from the abdominal aorta of rats in each group into K2-EDTA anticoagulant tubes. Plasma was separated by centrifugation and stored at −80 °C until analysis. Plasma levels of inflammatory cytokines and chemokines were measured using a RayPlex™ multiplex bead-based assay. The analytes included IFN-γ, IL-1β, IL-2, IL-10, IL-17, TNF-α, Eotaxin-1/CCL11, IL-2RA/CD25, IL-4, IL-6, IL-13, and KC/CXCL1.

The assay was performed according to the manufacturer’s instructions. Briefly, serial dilutions of the standards were prepared to generate calibration curves. Plasma samples were diluted at the recommended ratio and incubated with coded microspheres coated with analyte-specific capture antibodies for 2 h at room temperature. After washing, biotinylated detection antibodies and streptavidin–phycoerythrin (SA-PE) were sequentially added and incubated as instructed. The microspheres were then resuspended in 1× wash buffer and analyzed by flow cytometry. The APC channel was used for bead identification, whereas the PE channel was used for quantitative fluorescence detection. Analyte concentrations were calculated from the corresponding standard curves. Analytes with concentrations below the lower limit of detection in the majority of samples were excluded from subsequent statistical analyses.

### 2.6. Metabolomic Analysis of the Spinal Dorsal Horn

#### 2.6.1. Preparation of Spinal Dorsal Horn Samples

Based on the overall pharmacodynamic and functional evaluation, particularly the broader improvement in gait symmetry and ipsilateral hind-paw weight-bearing parameters observed in the high-dose group, rats from the Sham, Model, and high-dose LGZ + SIN groups were selected for subsequent metabolomic and transcriptomic analyses of the spinal dorsal horn. After completion of the behavioral assessments on treatment day 7, rats were anesthetized, and the lumbar enlargement of the spinal cord was rapidly removed. The L4–L6 spinal segments were identified according to anatomical landmarks, and the ipsilateral spinal dorsal horn was dissected. Tissue samples were processed under precooled conditions, immediately snap-frozen in liquid nitrogen, and subsequently stored at −80 °C until analysis. Repeated freeze–thaw cycles were avoided.

Approximately 20 mg of spinal dorsal horn tissue was accurately weighed and transferred into a 1.5-mL centrifuge tube. After the addition of 300 μL of prechilled methanol, the tissue was homogenized and sonicated for 2 min. The samples were then vortex-mixed and incubated at −20 °C for 2 h to facilitate protein precipitation and metabolite extraction. Following centrifugation at 4 °C, the supernatant was collected as the spinal dorsal horn extract. Of this extract, 4 μL was used for protein quantification using the bicinchoninic acid (BCA) assay, 50 μL was used for widely targeted metabolomic analysis, 10 μL was used for quantitative neurotransmitter analysis, and the remaining extract was used for oxylipin profiling. To minimize the effects of differences in tissue input and extraction efficiency on metabolite quantification, metabolomic data were further normalized to the protein content of each sample.

#### 2.6.2. LC–MS/MS-Based Metabolomic Analysis

Widely targeted metabolomic analysis was performed according to a previously established method developed by our group [14]. More than 600 metabolites representing over 15 metabolite classes, including organic acids, amino acids, biogenic amines, polyamines, nucleotides, vitamins, coenzymes, carbohydrates, and steroids, were analyzed in spinal dorsal horn samples and identified using an in-house metabolite database.

Chromatographic separation was performed using an ACQUITY UPLC BEH Amide column (2.1 × 50 mm, 1.7 μm; Waters, Milford, MA, USA) equipped with a corresponding guard column (2.1 × 5 mm, 1.7 μm; Waters, Milford, MA, USA). Mobile phase A consisted of 95% water and 5% acetonitrile containing 5 mM ammonium formate and 0.01% formic acid, whereas mobile phase B consisted of 95% acetonitrile and 5% water containing 5 mM ammonium formate and 0.01% formic acid. The gradient elution program was as follows: 0–2 min, 95% B; 2–4 min, 95–90% B; 4–6 min, 90% B; 6–9 min, 90–85% B; 9–12 min, 85% B; 12–15 min, 85–75% B; 15–16 min, 75% B; 16–18 min, 75–50% B; 18–20 min, 50% B; 20–22 min, 50–25% B; 22–24 min, 25% B; 24–25 min, 25–95% B; and 25–30 min, 95% B. The flow rate was 0.3 mL·min^−1^, the column temperature was maintained at 35 °C, the autosampler temperature was set at 4 °C, and the injection volume was 5 μL.

Mass spectrometric analysis was performed using an electrospray ionization source in both positive- and negative-ion modes. The curtain gas was set at 40 psi and the collision gas at 9 psi. The ion spray voltages were +5500 V in positive-ion mode and −4500 V in negative-ion mode. The ion source temperature was maintained at 550 °C, and both the nebulizer gas and auxiliary gas were set at 55 psi. Each metabolite was quantified by multiple reaction monitoring (MRM) using optimized precursor/product ion transitions and compound-specific mass spectrometric parameters.

#### 2.6.3. Metabolomic Data Processing and Differential Metabolite Screening

Chromatographic peak identification and peak-area integration were performed using MultiQuant 3.0.3 software, and the datasets acquired in positive- and negative-ion modes were subsequently combined. During the analytical run, one quality control (QC) sample was inserted after every 10 study samples to evaluate analytical stability and data reliability. Metabolites with a coefficient of variation (CV) < 50% in the QC samples were retained, and the resulting sample-by-variable data matrix was used for subsequent statistical analyses.

Principal component analysis (PCA) was first performed to evaluate the overall metabolic profiles and distribution patterns among groups. Orthogonal partial least-squares discriminant analysis (OPLS-DA) was subsequently used to further assess differences in metabolic profiles between groups. The performance of the OPLS-DA models was evaluated using R^2^Y and Q^2^, and permutation testing was performed to assess the risk of model overfitting.

Differential metabolites were identified using the following criteria: variable importance in projection (VIP) > 1, Student’s *t*-test *p* < 0.05, and fold change > 1.2 or <0.83. Differential metabolites were identified separately for the Model versus Sham comparison and the high-dose LGZ + SIN versus Model comparison. Metabolites shared between the two comparisons were further identified for subsequent integrated transcriptomic and metabolomic pathway analysis. Metabolic pathway enrichment analysis was performed using the Metware Cloud platform and MetaboAnalyst 6.0, with *p* < 0.05 considered indicative of significant pathway enrichment.

### 2.7. Transcriptomic Analysis of the Spinal Dorsal Horn

#### 2.7.1. Spinal Dorsal Horn Sample Collection and RNA Extraction

The spinal dorsal horn samples used for transcriptomic analysis were derived from the same experimental groups as those used for metabolomic analysis. Frozen spinal dorsal horn tissues were thoroughly ground in liquid nitrogen and lysed with QIAzol Lysis Reagent. After centrifugation, the supernatant was collected, mixed thoroughly with chloroform, and centrifuged to allow phase separation. The upper aqueous phase was transferred to a new tube, and an equal volume of isopropanol was added to precipitate RNA. The RNA pellet was washed with 75% ethanol, briefly air-dried, and dissolved in 0.1% DEPC-treated water. The extracted RNA was stored at −80 °C until further analysis. RNA concentration and purity were assessed using a NanoDrop spectrophotometer (Thermo Fisher Scientific, Waltham, MA, USA), and RNA integrity was evaluated using an Agilent Bioanalyzer (Santa Clara, CA, USA). Only RNA samples that met the quality requirements were used for subsequent library construction and sequencing.

#### 2.7.2. Library Construction, Sequencing, and Quality Control

RNA-sequencing libraries were prepared according to the standard procedures of the sequencing platform. Briefly, libraries were constructed using the SMART-Seq™ v4 Ultra Low Input RNA Kit (Takara Bio, Kusatsu, Shiga, Japan) for Sequencing. After library quantification and assessment of fragment-size distribution, qualified libraries were subjected to high-throughput sequencing on the MGI DNBSEQ platform.

The raw sequencing data were first subjected to quality assessment and subsequently filtered using fastp. Adapter-contaminated reads, low-quality reads, reads containing excessive proportions of ambiguous nucleotides (N), and reads below the minimum length threshold were removed to obtain high-quality clean reads. The clean reads were then aligned to the rat reference genome (Rattus norvegicus, mRatBN7.2), and the resulting mapped reads were used for subsequent gene expression quantification and differential expression analysis.

#### 2.7.3. Differentially Expressed Gene Screening and Functional Analysis

Gene-level count matrices were generated from the sequencing alignment results, and differential expression analysis was performed using DESeq2. Differentially expressed genes (DEGs) were identified using the criteria of an adjusted *p* value < 0.05 and a fold change > 1.2 or <0.83.

DEGs were identified separately for the Model versus Sham comparison and the high-dose LGZ + SIN versus Model comparison. Overlapping DEGs between the two comparisons were subsequently identified and used for integrated transcriptomic and metabolomic pathway analysis.

### 2.8. Integrated Transcriptomic and Metabolomic Pathway Analysis

To further identify key central metabolic pathways associated with the effects of combined ligustrazine and sinomenine treatment in CCI-induced neuropathic pain, differential metabolites identified by metabolomic analysis and overlapping DEGs identified by transcriptomic analysis were subjected to integrated pathway analysis.

The analysis was performed using the Joint Pathway Analysis module of MetaboAnalyst 6.0. Differential metabolites and DEGs were mapped to the corresponding metabolic pathways of the rat, and pathways were evaluated by integrating enrichment significance with pathway topology-based impact values. A *p* value < 0.05 was used as the primary criterion for pathway selection. Pathway impact values, together with the representation of differential metabolites and DEGs within each pathway, were further considered to identify key pathways exhibiting coordinated alterations at both the transcriptomic and metabolomic levels.

### 2.9. Oxylipin Profiling of the Spinal Dorsal Horn

#### 2.9.1. Sample Preparation for Oxylipin Profiling

The spinal dorsal horn samples used for oxylipin profiling were derived from the same experimental groups as those used for metabolomic and transcriptomic analyses. Sample preparation was performed with reference to the method described by [19].

Oasis HLB solid-phase extraction cartridges (1 cc/30 mg; Waters, Milford, MA, USA) were sequentially conditioned with 1 mL methanol, 1 mL ethyl acetate, 1 mL methanol, and 2 mL water. An aliquot of 200 μL of the spinal dorsal horn extract described above was diluted with 1.8 mL of water containing 1% formic acid and loaded onto the cartridge. The original sample tube was rinsed with 200 μL of 10% methanol in water, and the rinse was also loaded onto the cartridge. The cartridge was then sequentially washed with 3 mL of water and 1 mL of 10% methanol in water to remove interfering components. After vacuum drying, the analytes were eluted with 0.5 mL acetonitrile followed by 1.5 mL ethyl acetate.

The eluate was dried to completeness by vacuum centrifugation at 10 °C and 1500 rpm. The residue was reconstituted in 50 μL methanol, vortex-mixed, and centrifuged at 20,000× *g* for 10 min. Subsequently, 45 μL of the supernatant was mixed with 5 μL of the internal standard mixture and subjected to LC–MS/MS analysis. Quantitative oxylipin data were further normalized to the protein content of each sample.

#### 2.9.2. Quantitative LC–MS/MS Analysis of Oxylipins

Quantitative oxylipin analysis was performed based on a previously reported method with modifications optimized for the present study [19]. The analytical panel covered 172 polyunsaturated fatty acids (PUFAs) and oxylipins generated through cyclooxygenase (COX), lipoxygenase (LOX), and cytochrome P450 (CYP450) pathways. The internal standard mixture consisted of AA-d8 (10 μg/mL), 20-HETE-d6 (1 μg/mL), leukotriene B4-d4 (1 μg/mL), and PGE2-d5 (1 μg/mL). AA-d8 was used for normalization of PUFAs, whereas 20-HETE-d6, leukotriene B4-d4, and PGE2-d5 were used as internal standards for metabolites associated with the CYP450, LOX, and COX pathways, respectively.

Mixed standard solutions were serially diluted with methanol to prepare calibration standards ranging from 1 to 200 ng/mL. Low-, medium-, and high-concentration quality control (QC) samples were prepared at 1.25, 10, and 80 ng/mL, respectively, for method validation. Quantification was based on the peak-area ratio of each analyte to its corresponding internal standard. The correlation coefficients of the calibration curves for all analytes were >0.99. The relative standard deviations for both intra-day and inter-day precision were <10%, and the accuracy deviations of the low-, medium-, and high-concentration QC samples were within 15%, indicating that the method was suitable for quantitative oxylipin analysis in biological samples.

Chromatographic separation was performed using a BEH C18 column (2.1 × 100 mm, 1.7 μm; Waters, Milford, MA, USA). Mobile phase A consisted of water containing 0.1% formic acid, whereas mobile phase B consisted of acetonitrile/isopropanol (90:10, *v*/*v*) containing 0.1% formic acid. The gradient elution program was as follows: 0–1.0 min, 25% B; 1.0–2.0 min, 25–35% B; 2.0–10.0 min, 35–50% B; 10.0–20.0 min, 50–65% B; 20.0–25.0 min, 65–95% B; 25.0–27.0 min, 95% B; 27.0–27.1 min, 95–25% B; and 27.1–30.0 min, 25% B. The flow rate was 0.3 mL·min^−1^, the column temperature was maintained at 45 °C, the autosampler temperature was set at 10 °C, and the injection volume was 2 μL.

Mass spectrometric detection was performed using an electrospray ionization source in negative-ion mode with multiple reaction monitoring (MRM). The curtain gas was set at 35 psi and the collision gas at 9 psi. The ion spray voltage was 4500 V, the ion source temperature was maintained at 550 °C, and both the nebulizer gas and auxiliary gas were set at 50 psi. Compound-specific mass spectrometric parameters, including the precursor/product ion transitions, declustering potential, and collision energy, were individually optimized for each oxylipin to achieve high sensitivity and analytical stability. Representative chromatograms are shown in Figures S2–S6.

#### 2.9.3. Construction of an In-House Oxylipin Pathway Database and Pathway Enrichment Analysis

To comprehensively characterize the oxylipin metabolic network in the spinal dorsal horn, an in-house oxylipin pathway database (Version 1.0) was constructed based on metabolic reaction relationships and enzymatic information for PUFAs and their derived oxylipins curated from public databases, including Rhea and Reactome. The database covered both ω-6 and ω-3 PUFA families, including linoleic acid (LA), γ-linolenic acid (GLA), dihomo-γ-linolenic acid (DGLA), arachidonic acid (AA), α-linolenic acid (ALA), eicosapentaenoic acid (EPA), docosahexaenoic acid (DHA), and their downstream oxylipins. Compared with the relatively limited coverage of oxylipin-related pathways in the KEGG database, the in-house database further incorporated detailed metabolic branches of GLA, DGLA, EPA, and DHA mediated by the COX, LOX, and CYP450 pathways.

The database comprised seven major PUFAs and more than 160 related metabolites, encompassing seven major metabolic pathways and 15 detailed metabolic branches mediated by COX, LOX, and CYP450 enzymes. After identification of differential oxylipins, pathway mapping and enrichment analyses were performed separately using the KEGG database and the in-house oxylipin pathway database, followed by pathway enrichment analysis using the MetaboAnalyst platform. A *p* value < 0.05 was considered indicative of significant pathway enrichment.

### 2.10. Analysis of Arginine–Polyamine Metabolism and Redox-Related Indicators

To further validate the arginine-related metabolic pathways identified by integrated metabolomic and transcriptomic analyses, LC–MS/MS was used to quantify metabolites associated with arginine–polyamine metabolism and redox homeostasis in the spinal dorsal horn. Spinal dorsal horn tissues from the Sham, Model, and high-dose LGZ + SIN groups were analyzed.

The arginine-related metabolic panel included arginine, L-ornithine, putrescine, spermine, spermidine, N1-acetylspermine, urea, guanidinoacetate, creatine, agmatine, and citrulline. These metabolites were used to assess alterations in metabolic branches involving ornithine–polyamine metabolism, creatine metabolism, agmatine metabolism, and citrulline metabolism. In addition, metabolite ratios, including the N1-acetylspermine/spermine ratio, were calculated to reflect the status of polyamine acetylation and catabolism.

Redox-related indicators included NADP, NADPH, GSSG, GSH, glutamine, glutamate, cystine, and cysteine. The NADP/NADPH, GSH/GSSG, glutamine/glutamate, and cystine/cysteine ratios were further calculated to evaluate redox homeostasis, glutathione redox status, glutamine–glutamate metabolic balance, and cystine–cysteine homeostasis in the spinal dorsal horn, respectively. Sample preparation and LC–MS/MS analysis were performed according to the widely targeted metabolomic procedure described above. Each metabolite was quantified using optimized ion transitions and mass spectrometric parameters, and the resulting data were normalized to the protein content of each sample.

### 2.11. Targeted Neurotransmitter Analysis of the Spinal Dorsal Horn

#### 2.11.1. Sample Preparation and Benzoyl Chloride Derivatization

The spinal dorsal horn samples used for targeted neurotransmitter analysis were derived from the same experimental groups as those used for the omics analyses described above. Sample preparation was performed using a benzoyl chloride derivatization method established previously by our group [14].

Briefly, 10 μL of the spinal dorsal horn extract was sequentially mixed with 10 μL of 100 mM sodium tetraborate buffer and 10 μL of 1% benzoyl chloride in acetonitrile. The mixture was vortexed for 5 min and then allowed to react for 5 min at 25 °C in the dark. After derivatization, the samples were centrifuged at 20,000× *g* for 10 min at 25 °C. Subsequently, 24 μL of the supernatant was mixed with 6 μL of an isotopically labeled internal standard solution and subjected to LC–MS/MS analysis. The internal standard solution was freshly prepared by derivatizing a mixed standard solution (100–1000 ng/mL) with d5-benzoyl chloride using the same procedure.

#### 2.11.2. Quantitative LC–MS/MS Analysis of Neurotransmitters

Based on a previously established method from our group [14], targeted quantitative analysis was performed for 44 metabolites involved in major neurotransmitter-related metabolic axes in the spinal dorsal horn, including tryptophan–serotonin/kynurenine/indole metabolism, phenylalanine–tyrosine–catecholamine metabolism, glutamine–glutamate–γ-aminobutyric acid metabolism, and choline/acetylcholine-related pathways.

Chromatographic separation was performed using a PFP C18 column (2.1 × 50 mm, 1.8 μm; Waters, Milford, MA, USA). Mobile phase A consisted of water containing 5 mM ammonium formate and 0.1% formic acid, whereas mobile phase B was acetonitrile. The gradient elution program was as follows: 0–1.0 min, 20% B; 1.0–2.0 min, 20–50% B; 2.0–6.0 min, 50–80% B; 6.0–6.5 min, 80–95% B; 6.5–8.0 min, 95% B; 8.0–8.1 min, 95–20% B; and 8.1–10.0 min, 20% B. The flow rate was 0.3 mL·min^−1^, the column temperature was maintained at 35 °C, the autosampler temperature was set at 4 °C, and the injection volume was 2 μL.

Mass spectrometric detection was performed using an electrospray ionization source in positive-ion mode with MRM mode. The curtain gas was set at 35 psi and the collision gas at 9 psi. The ion spray voltage was 5500 V, the ion source temperature was maintained at 550 °C, and both the nebulizer gas and auxiliary gas were set at 55 psi. Quantification of each analyte was performed using optimized precursor/product ion transitions, declustering potentials, and collision energies.

Calibration curves were generated using serial dilutions of mixed standards. Linear regression was performed using the peak-area ratio of each analyte to its corresponding internal standard as the dependent variable and analyte concentration as the independent variable. Concentrations of neurotransmitters and related metabolites in biological samples were calculated from the corresponding calibration curves and normalized to the protein content of each sample.

#### 2.11.3. Analysis of Neurotransmitter Metabolic Ratios and Homeostasis

To further characterize the effects of CCI and combined ligustrazine and sinomenine treatment on neurotransmitter metabolic activity and homeostatic balance in the spinal dorsal horn, several metabolite ratios were calculated in addition to the concentrations of individual metabolites.

Within the monoaminergic neurotransmitter system, (DOPAC + HVA)/DA, 3-MT/DA, and HVA/DA were used as indices of dopamine turnover. NAS/5-HT and Mel/5-HT were used to assess downstream serotonin metabolic activity, whereas NM/NE was used to reflect norepinephrine-related metabolic changes.

Within the excitatory and inhibitory amino acid systems, the Glu/GABA ratio was used to evaluate the balance between excitatory and inhibitory neurotransmission, whereas the Gln/Glu ratio was used to assess the glutamine–glutamate cycle and neuron–glia metabolic coupling.

Within the kynurenine pathway, the Kyn/Trp ratio was used to reflect the relative conversion of tryptophan toward the kynurenine pathway, whereas KA/Kyn, QA/Kyn, and KA/QA were used to evaluate the relative metabolic distribution among downstream branches of the kynurenine pathway. Collectively, these ratios were used to characterize neurotransmitter turnover, excitatory/inhibitory balance, and kynurenine pathway homeostasis in the spinal dorsal horn.

### 2.12. qRT-PCR Analysis

Quantitative real-time PCR (qRT-PCR) was performed to determine the mRNA expression levels of genes associated with polyamine metabolism, redox homeostasis, and central sensitization in the spinal dorsal horn. Ipsilateral L4–L6 spinal dorsal horn tissues were collected from rats in each group, and total RNA was extracted. RNA concentration and purity were assessed using a NanoDrop spectrophotometer. Total RNA was then reverse-transcribed into cDNA according to the manufacturer’s instructions for the cDNA reverse-transcription kit. The reverse-transcription conditions were 37 °C for 15 min, 50 °C for 5 min, and 98 °C for 5 min, followed by storage at 4 °C.

Real-time PCR was performed using SYBR Green real-time PCR master mix. The amplification protocol consisted of an initial denaturation at 95 °C for 30 s, followed by 40 cycles of 95 °C for 5 s, 55 °C for 10 s, and 72 °C for 15 s. Melting-curve analysis was performed after amplification to confirm the specificity of the PCR products. The target genes included SAT1, SLC7A11/xCT, and GRIN2B, with ACTB used as the reference gene. Relative mRNA expression was calculated using the 2^−ΔΔCt^ method. Primer sequences are listed in Table S1.

### 2.13. Western Blot Analysis

Western blotting was performed to determine the protein expression levels of SAT1, xCT, and NR2B in the spinal dorsal horn. Ipsilateral L4–L6 spinal dorsal horn tissues from each group were thawed on ice and lysed in pre-chilled RIPA lysis buffer supplemented with protease and phosphatase inhibitors. After thorough homogenization, the samples were centrifuged at 12,000× *g* for 15 min at 4 °C, and the supernatants were collected. Protein concentrations were determined using the BCA assay, after which all samples were adjusted to an equal protein concentration. Protein loading buffer was then added, and the samples were denatured at 100 °C for 15 min.

Equal amounts of protein were separated by SDS-PAGE and transferred onto membranes. After blocking at room temperature, the membranes were incubated with primary antibodies against SAT1, xCT, or NR2B. Following washing, the membranes were incubated with HRP-conjugated secondary antibodies or processed using a one-step rapid Western blot detection kit. Protein bands were visualized using enhanced chemiluminescence (ECL), and images were acquired for densitometric analysis using ImageJ 1.53 software. SAT1 and xCT protein expression levels were normalized to GAPDH, whereas NR2B protein expression was normalized to β-actin.

### 2.14. Statistical Analysis

Statistical analyses and graph generation were performed using GraphPad Prism8.0. Quantitative data are presented as the mean ± standard error of the mean (SEM). Comparisons between two groups were performed using Student’s *t*-test, whereas comparisons among multiple groups were performed using one-way analysis of variance (ANOVA). For behavioral data involving measurements at multiple time points, repeated-measures ANOVA was used where appropriate.

For metabolomic analysis, oxylipin profiling, and targeted neurotransmitter analysis, differential metabolites were identified according to the criteria specified in the corresponding methodological sections. A *p* value < 0.05 was considered statistically significant.

## 3. Results

### 3.1. Combined Ligustrazine and Sinomenine Treatment Ameliorates Pain-Related Behaviors and Gait Dysfunction in CCI Rats

#### 3.1.1. Combined Ligustrazine and Sinomenine Treatment Increases the Mechanical Withdrawal Threshold and Attenuates Cold Hypersensitivity in CCI Rats

As a general indicator of postoperative condition, body weight gain was monitored throughout the experimental period. Although all groups showed progressive weight gain, the Model group exhibited significantly lower body weight than the Sham group following CCI surgery (Figure S1). Combined ligustrazine and sinomenine treatment partially restored body weight gain, with the greatest improvement observed in the high-dose group.

MWT testing showed that CCI induced persistent mechanical hypersensitivity, as evidenced by significantly reduced MWTs in the Model group compared with the Sham group on treatment days 1, 3, and 7 (Figure 1A). Following combined ligustrazine and sinomenine treatment, MWTs showed varying degrees of improvement during the treatment period; however, on treatment day 7, a statistically significant increase compared with the Model group was observed only in the medium-dose group. These findings indicate that the effect on mechanical hypersensitivity was not strictly dose-dependent under the present experimental conditions.

Similarly, CCI markedly increased cold hypersensitivity scores compared with the Sham group (Figure 1B). Combined ligustrazine and sinomenine treatment produced downward trends in cold hypersensitivity scores across the treatment groups, particularly on treatment days 3 and 7; however, these differences did not reach statistical significance compared with the Model group. Collectively, these results suggest that combined ligustrazine and sinomenine treatment improves mechanical hypersensitivity and shows a tendency to attenuate cold hypersensitivity in CCI rats.

#### 3.1.2. Combined Ligustrazine and Sinomenine Treatment Improves Ipsilateral Hind-Paw Weight Bearing and Gait Symmetry in CCI Rats

To further evaluate the effects of combined ligustrazine and sinomenine treatment on pain-related motor dysfunction in CCI rats, gait analysis was performed to assess hind-paw footprints, plantar pressure distribution, and gait symmetry. Footprint images showed that rats in the Sham group exhibited comparable footprint sizes between the two hind paws, with a relatively balanced pressure distribution (Figure 1C). In contrast, rats in the Model group showed a markedly reduced footprint size and contact area of the ipsilateral hind paw, together with increased footprint intensity of the contralateral hind paw, indicating reduced weight bearing on the injured side and compensatory loading of the contralateral hind paw following CCI. After combined ligustrazine and sinomenine treatment, both the footprint pattern and ground-contact pressure of the ipsilateral hind paw were restored to varying degrees, with the most pronounced improvement observed in the high-dose group.

The pressure–time curves further demonstrated that the Sham group displayed relatively regular pressure profiles for both hind paws, with largely symmetrical bilateral weight bearing (Figure 1D). In the Model group, the pressure profile of the ipsilateral hind paw was markedly attenuated, as reflected by reduced contact intensity, shortened contact duration, and an imbalanced pressure distribution between the two hind paws. Following combined ligustrazine and sinomenine treatment, the pressure profile of the ipsilateral hind paw improved to varying degrees across the treatment groups. The low-dose group showed only limited recovery, whereas the medium-dose group exhibited an increased ipsilateral pressure profile accompanied by prolonged contact duration. In the high-dose group, the ipsilateral pressure profile became more robust, with both contact intensity and duration approaching those of the contralateral hind paw. Pregabalin treatment also improved ipsilateral pressure distribution, although the overall extent of recovery was slightly less pronounced than that observed in the high-dose LGZ + SIN group.

As shown in Table 1, the ipsilateral-to-contralateral hind-paw ratios for most gait symmetry parameters in the Sham group were close to 100%, indicating relatively symmetrical gait distribution between the two hind limbs under normal conditions. Compared with the Sham group, the Model group showed significant decreases in the LH/RH ratios for stance duration, stance phase ratio, single-hind-paw support duration, and maximum contact area, together with a significant increase in the LH/RH ratio for swing duration. These findings indicate that CCI resulted in insufficient support and reduced weight bearing of the ipsilateral hind paw, accompanied by prolonged pain-avoidance-related swing duration. Specifically, in the Model group, the LH/RH ratio for stance duration decreased to 70.2% ± 6.1%, the stance phase ratio to 70.3% ± 5.9%, the single-hind-paw support duration to 64.4% ± 4.7%, and the maximum contact area to 53.1% ± 9.7%, whereas the swing duration ratio increased to 216.7% ± 22.6%.

Compared with the Model group, the low-dose LGZ + SIN group showed trends toward improvement in multiple gait parameters, although the differences did not reach statistical significance. The medium-dose group showed significant increases in the LH/RH ratios for stance phase, single-hind-paw support duration, and maximum contact area, indicating improved support and weight-bearing function of the ipsilateral hind paw. The high-dose group exhibited a broader improvement in gait performance, with significant increases in the LH/RH ratios for stance duration, stance phase, single-hind-paw support duration, and maximum contact area, together with a reduction in the abnormally elevated swing duration ratio. Pregabalin primarily improved the LH/RH ratios for stance duration, stance phase, and single-hind-paw support duration, whereas its effects on swing duration and maximum contact area were comparatively limited. Collectively, these results indicate that combined ligustrazine and sinomenine treatment ameliorates reduced ipsilateral hind-paw loading, impaired support, and gait asymmetry in CCI rats, with the high-dose treatment producing the most comprehensive improvement in gait dysfunction.

### 3.2. Combined Ligustrazine and Sinomenine Treatment Modulates Systemic Inflammatory Dysregulation in CCI Rats

The improvement in neuropathic pain-related behaviors following combined ligustrazine and sinomenine treatment may be closely associated with restoration of peripheral inflammatory mediator homeostasis. Plasma levels of IFN-γ, IL-1β, IL-2, IL-10, IL-17, TNF-α, Eotaxin-1/CCL11, IL-4, IL-13, and KC/CXCL1. IL-6 and IL-2RA/CD25 were excluded from subsequent analyses because their concentrations were below the lower limit of detection in the majority of samples. To visualize overall differences in inflammatory mediator profiles among the experimental groups, the levels of individual analytes were Z-score normalized, and heatmaps with hierarchical clustering were generated based on the mean Z-scores for each group.

As shown in Figure 1E, the plasma inflammatory mediator profiles of the Sham and Model groups exhibited clearly distinct distribution patterns. Compared with the Sham group, the Model group showed overall increases in IL-17, TNF-α, IL-2, IFN-γ, and IL-1β, with particularly pronounced elevations in IL-17 and TNF-α. In contrast, Eotaxin-1, IL-4, IL-10, KC, and IL-13 generally showed lower levels in the Model group. Hierarchical clustering further revealed that IL-17, TNF-α, IL-2, IFN-γ, and IL-1β were predominantly grouped into one cluster, whereas Eotaxin-1, IL-4, IL-10, KC, and IL-13 formed a separate cluster. These findings indicate that CCI induces a distinct systemic inflammatory profile characterized by concomitant increases in pro-inflammatory mediators and decreases in immunoregulatory mediators.

Following combined ligustrazine and sinomenine treatment, the relative expression patterns of multiple CCI-associated inflammatory mediators were restored to varying degrees. In the low-dose group, IL-17, TNF-α, IFN-γ, and IL-1β were reduced relative to the Model group, whereas Eotaxin-1, IL-4, IL-10, and IL-13 showed trends toward restoration, suggesting that low-dose treatment modulated both pro-inflammatory and immunoregulatory mediators. In the medium-dose group, IL-17, TNF-α, IL-1β, and IFN-γ were also reduced relative to the Model group, while KC and IL-13 showed more evident shifts toward the Sham profile; IL-4 and IL-10 likewise exhibited partial recovery. Consistent with the statistical analysis of the original concentration data, the medium-dose treatment exerted relatively broad regulatory effects on multiple mediators, including IL-17, IL-1β, TNF-α, IL-4, and IL-13.

CCI-induced neuropathic pain was accompanied by systemic inflammatory imbalance, characterized by altered expression patterns of pro-inflammatory and immunoregulatory mediators. Combined ligustrazine and sinomenine treatment partially restored this inflammatory profile, suggesting that peripheral inflammatory modulation may contribute to its analgesic effects. However, given that neuropathic pain maintenance is primarily associated with central sensitization, subsequent analyses focused on metabolic remodeling and molecular alterations within the spinal dorsal horn.

### 3.3. Combined Ligustrazine and Sinomenine Treatment Reshapes the Transcriptomic and Metabolomic Profiles of the Spinal Dorsal Horn in CCI Rats

#### 3.3.1. Global Metabolomic Changes and Screening of Differential Metabolites in the Spinal Dorsal Horn

Widely targeted metabolomic analysis was first performed to characterize alterations in the metabolic profile of the spinal dorsal horn across the experimental groups. Principal component analysis (PCA) showed that the first and second principal components accounted for 25.78% and 15.42% of the total variance, respectively (Figure 2A). The samples from different groups exhibited a certain degree of separation in their overall metabolic profiles, suggesting that both CCI and combined ligustrazine and sinomenine treatment induced alterations in the metabolic state of the spinal dorsal horn.

Orthogonal partial least-squares discriminant analysis (OPLS-DA) was subsequently performed to further evaluate between-group differences in metabolic profiles (Figure 2B). For the Model versus Sham comparison, the OPLS-DA model yielded R^2^X, R^2^Y, and Q^2^ values of 0.467, 0.994, and 0.485, respectively. For the LGZ + SIN versus Model comparison, the corresponding R^2^X, R^2^Y, and Q^2^ values were 0.663, 0.999, and 0.701, respectively, indicating good discriminatory performance of the models. Permutation testing showed no evidence of obvious model overfitting (Figure 2C), supporting the robustness and reliability of the metabolomic analysis.

Differential metabolites were then screened using the criteria of VIP > 1, *p* < 0.05, and fold change > 1.2 or <0.83. Compared with the Sham group, 35 differential metabolites were identified in the Model group, including 23 upregulated and 12 downregulated metabolites. Compared with the Model group, 54 differential metabolites were identified in the LGZ + SIN group, including 14 upregulated and 40 downregulated metabolites. Volcano plots and hierarchical clustering heatmaps further demonstrated marked alterations in the spinal dorsal horn metabolite profile following CCI, whereas combined ligustrazine and sinomenine treatment partially reversed a subset of these abnormal metabolic changes (Figure 2D–E).

To identify core metabolites associated with both the pathological state induced by CCI and the response to pharmacological intervention, Venn analysis was performed on the differential metabolites identified in the Model versus Sham and LGZ + SIN versus Model comparisons (Figure 2F). Six overlapping differential metabolites were identified between the two comparisons, suggesting that these metabolites may represent core metabolic alterations associated with both CCI-induced metabolic disturbances and the response to combined ligustrazine and sinomenine treatment. Detailed information on these overlapping metabolites, including metabolite annotation, database identifiers, fold changes, and statistical parameters, is provided in Supplementary Table S2.

#### 3.3.2. Combined Ligustrazine and Sinomenine Treatment Modulates Transcriptional Profiles and the Functional Characteristics of Differentially Expressed Genes in the Spinal Dorsal Horn of CCI Rats

To characterize CCI-induced molecular alterations in the spinal dorsal horn at the transcriptional level and to evaluate the regulatory effects of combined ligustrazine and sinomenine treatment, transcriptomic sequencing and bioinformatic analyses were performed on spinal dorsal horn tissues from the Sham, Model, and high-dose LGZ + SIN groups.

Principal component analysis was first performed to assess the overall distribution of gene expression profiles among the experimental groups. As shown in Figure 3A, the first and second principal components accounted for 21.99% and 15.47% of the total variance, respectively, together explaining 37.46% of the overall transcriptional variation. Samples from the Sham, Model, and LGZ + SIN groups showed distinct distribution trends in the PCA score plot, although the confidence ellipses of the three groups overlapped to varying degrees. These findings indicate that both CCI and combined ligustrazine and sinomenine treatment altered the overall transcriptional profile of the spinal dorsal horn.

Differential gene expression analysis was subsequently performed. As shown in Figure 3B, 659 differentially expressed genes (DEGs) were identified in the Model group compared with the Sham group, including 393 upregulated and 266 downregulated genes, indicating that CCI induced extensive transcriptional alterations in the spinal dorsal horn. In comparison with the Model group, 130 DEGs were identified in the LGZ + SIN group, including 74 upregulated and 56 downregulated genes, suggesting that combined ligustrazine and sinomenine treatment modulated a subset of the CCI-associated transcriptional abnormalities. The relatively smaller number of DEGs induced by treatment compared with the broader transcriptional changes caused by CCI further suggests that the effects of the combination may be concentrated within specific molecular regulatory networks.

To identify candidate genes associated with both CCI pathology and the response to pharmacological intervention, Venn analysis was performed using DEGs from the Model versus Sham and LGZ + SIN versus Model comparisons. As shown in Figure 3C, 625 DEGs were unique to the Model versus Sham comparison, whereas 96 DEGs were unique to the LGZ + SIN versus Model comparison. A total of 34 DEGs were shared between the two comparisons. Detailed information on these overlapping DEGs, including gene IDs, gene symbols, fold changes, and *p* values for both comparisons, is provided in Supplementary Table S3. Because these overlapping genes were altered following CCI and also responded to combined ligustrazine and sinomenine treatment, they may represent candidate molecular mediators linking CCI-associated pathological changes with the regulatory effects of the combination treatment.

Clustering analysis of the expression patterns of the 34 overlapping DEGs revealed heterogeneous transcriptional responses across the Sham, Model, and LGZ + SIN groups (Figure 3D). Some genes were markedly upregulated or downregulated in the Model group relative to the Sham group and showed varying degrees of normalization toward the Sham profile following LGZ + SIN treatment, whereas others exhibited treatment-specific expression patterns. These findings indicate that combined ligustrazine and sinomenine treatment did not uniformly reverse all CCI-associated transcriptional alterations but instead selectively modulated a subset of genes. Among the overlapping DEGs, candidate genes such as Nos1, which is involved in neuronal signaling and metabolic regulation, provided a basis for subsequent integrated transcriptomic and metabolomic analyses.

KEGG functional annotation showed that the overlapping DEGs were associated with multiple categories of pathways related to metabolism, cellular processes, organismal systems, and disease-related functions (Figure 3E). Metabolism-related pathways included purine metabolism, nucleotide metabolism, arginine biosynthesis, arginine and proline metabolism, and pathways associated with other drug-metabolizing enzymes. The overlapping DEGs were also annotated to calcium signaling, cytokine–cytokine receptor interaction, peroxisome-related processes, and antigen processing and presentation. These findings suggest that genes jointly affected by CCI and combined ligustrazine and sinomenine treatment may participate in metabolic regulation, immune-inflammatory responses, and cellular signal transduction.

Gene Ontology (GO) functional annotation further showed that, within the biological process category, the overlapping DEGs were primarily associated with cellular processes, biological regulation, response to stimulus, immune system processes, and developmental processes (Figure 3F). Within the cellular component category, the genes were mainly annotated to cellular anatomical entities and protein-containing complexes. Within the molecular function category, the major annotations included binding activity, catalytic activity, transcription regulator activity, and molecular function regulator activity. Collectively, these results suggest that the overlapping DEGs may contribute to CCI-associated abnormalities in the spinal dorsal horn and to the effects of combined ligustrazine and sinomenine treatment through coordinated involvement in stimulus responses, immune regulation, metabolic processes, and molecular interactions.

#### 3.3.3. Integrated Transcriptomic and Metabolomic Analysis Reveals Alterations in Arginine-Related Metabolic Networks

To further identify key biological pathways showing coordinated alterations at both the transcriptional and metabolic levels, 34 overlapping differentially expressed genes and 83 union differential metabolites were subjected to integrated transcriptomic and metabolomic pathway analysis using MetaboAnalyst 6.0.

As shown in Figure 4A, the identified metabolic pathways differed substantially in both enrichment significance and pathway impact. In the Joint Pathway Analysis bubble plot, arginine and proline metabolism exhibited both appreciable enrichment significance and pathway impact, while arginine biosynthesis was also enriched with both key metabolites and genes. These findings suggest that arginine-related metabolic networks may represent an important point of convergence between transcriptional and metabolic alterations.

Further analysis of the molecules mapped to arginine-related pathways showed that, within the arginine and proline metabolism pathway, the matched DEG was NOS1, whereas the matched metabolites included putrescine, spermine, and 4-aminobutanoate (GABA). Within the arginine biosynthesis pathway, Nos1 was again identified as the matched DEG, together with N-acetyl-L-glutamate as the matched metabolite. These findings suggest that NOS1 may represent an important molecular node linking alterations in arginine metabolism with transcriptional dysregulation, whereas N-acetyl-L-glutamate, putrescine, spermine, and 4-aminobutanoate reflect alterations in arginine precursor synthesis, ornithine–polyamine metabolism, and related downstream metabolic branches.

To further illustrate the positions of the differential genes and metabolites within the arginine-related metabolic network, the arginine biosynthesis and arginine and proline metabolism pathways were locally integrated, as shown in Figure 4B. The two pathways were connected through L-arginine as a central metabolic node. In the arginine biosynthesis branch, L-glutamate is converted to L-arginine through intermediates including N-acetyl-L-glutamate, L-ornithine, and L-citrulline, among which N-acetyl-L-glutamate was identified as a differential metabolite. In the arginine and proline metabolism branch, L-arginine can be converted to nitric oxide and L-citrulline through a reaction involving NOS1. Alternatively, L-arginine can be converted to L-ornithine via ARG1 and subsequently enter the polyamine biosynthetic pathway through ODC1, leading sequentially to the formation of putrescine, spermidine, and spermine.

Collectively, the integrated transcriptomic and metabolomic analyses demonstrated that molecular abnormalities in the spinal dorsal horn of CCI rats involved arginine-related metabolic networks. Both arginine and proline metabolism and arginine biosynthesis showed coordinated mapping of differential genes and metabolites, with NOS1, N-acetyl-L-glutamate, putrescine, spermine, and GABA representing key differential nodes within this network. These findings suggest that disturbances in arginine biosynthesis and in the metabolic allocation of arginine toward downstream branches involving nitric oxide, polyamines, and GABA may contribute to CCI-induced metabolic remodeling in the spinal dorsal horn and may constitute an important metabolic basis for the central regulatory effects of combined ligustrazine and sinomenine treatment.

### 3.4. Combined Ligustrazine and Sinomenine Treatment Ameliorates Oxylipin Metabolic Disturbances in the Spinal Dorsal Horn of CCI Rats

#### 3.4.1. Quantitative Oxylipin Analysis and Screening of Differential Oxylipins in the Spinal Dorsal Horn

Oxylipin profiling was performed on spinal dorsal horn samples from each experimental group. Among the 172 targeted analytes included in the quantitative panel, a total of 49 polyunsaturated fatty acids (PUFAs) and their derived oxylipins were detected (Figure 5A). The quantitative data were normalized to the protein content of each sample and subsequently used for differential oxylipin screening and pathway analysis.

Comparisons of Model versus Sham and LGZ + SIN versus Model identified a total of 11 differential oxylipins. Pathway enrichment analyses were subsequently performed using both the KEGG database and the in-house oxylipin pathway database (Figure 5B). Compared with the KEGG database, the in-house database identified three additional enriched metabolic pathways, providing broader coverage of oxylipin metabolic alterations associated with neuropathic pain.

#### 3.4.2. CCI Induces Alterations in DHA-, EPA-, and AA-Related Oxylipin Metabolism

In the Model versus Sham comparison, a total of nine differential oxylipins were identified, including linoleic acid, docosahexaenoic acid (DHA), eicosapentaenoic acid (EPA), (±)-13-HDoHE, (±)-20-HDoHE, and 19(20)-EpDPE. These differential metabolites were primarily enriched in linoleic acid/α-linolenic acid metabolism, DHA metabolism, EPA metabolism, and arachidonic acid metabolism, indicating that CCI induced marked alterations in PUFA- and oxylipin-related metabolic networks in the spinal dorsal horn.

Further analysis showed that DHA levels were significantly decreased, whereas EPA levels were significantly increased in the spinal dorsal horn of CCI rats, indicating an altered metabolic state of ω-3 PUFA precursors. Meanwhile, the DHA-derived oxylipin (±)-20-HDoHE and the EPA-derived oxylipin 19(20)-EpDPE were both abnormally elevated, suggesting that CCI affected not only the levels of PUFA precursors but also the profiles of their downstream oxylipin products (Figure 5C).

Collectively, these findings indicate that oxylipin metabolic disturbances in the spinal dorsal horn under CCI-induced neuropathic pain are not restricted to changes in individual metabolites but involve multiple PUFA-related pathways, including those associated with DHA, EPA, AA, and LA/ALA. These results suggest systemic remodeling of the local lipid mediator metabolic network.

#### 3.4.3. Combined Ligustrazine and Sinomenine Treatment Restores Key DHA/EPA-Related Oxylipin Alterations

Further comparison of the LGZ + SIN and Model groups showed that multiple CCI-induced abnormal oxylipins were restored to varying degrees following combined ligustrazine and sinomenine treatment. Among the affected pathways, DHA metabolism and EPA metabolism were particularly responsive to treatment, suggesting that the combination modulated disturbances in ω-3 PUFA-related oxylipin metabolism.

At the individual metabolite level, the abnormally elevated levels of (±)-20-HDoHE and 19(20)-EpDPE observed in the Model group were markedly reduced following combined ligustrazine and sinomenine treatment (Figure 5D), indicating that the treatment attenuated the CCI-induced accumulation of downstream DHA- and EPA-derived oxylipins. In parallel, the levels of precursor metabolites, including DHA and EPA, also showed trends toward normalization. These findings suggest that combined ligustrazine and sinomenine treatment may ameliorate local oxylipin metabolic disturbances in the spinal dorsal horn by modulating DHA/EPA-related metabolic flux.

Overall, oxylipin profiling demonstrated that CCI induced widespread alterations in PUFA and oxylipin metabolic networks in the spinal dorsal horn, predominantly involving DHA-, EPA-, AA-, and LA/ALA-related pathways. Combined ligustrazine and sinomenine treatment partially restored key DHA/EPA-related oxylipin abnormalities, particularly by reducing the elevated levels of (±)-20-HDoHE and 19(20)-EpDPE. These findings indicate that the combination exerts a regulatory effect on lipid mediator metabolic disturbances in the spinal dorsal horn.

### 3.5. Combined Ligustrazine and Sinomenine Treatment Modulates Neurotransmitter Metabolic Homeostasis in the Spinal Dorsal Horn of CCI Rats

#### 3.5.1. Global Changes in the Targeted Neurotransmitter Metabolic Profile and Pathway Enrichment Analysis of Differential Metabolites

Principal component analysis was first performed as an unsupervised approach to characterize the overall neurotransmitter metabolic profiles across the experimental groups. As shown in Figure S7A, the Sham and Model groups exhibited a degree of separation in their overall neurotransmitter metabolic profiles, indicating that CCI altered neurotransmitter metabolism in the spinal dorsal horn. Compared with the Model group, samples from the low-, medium-, and high-dose LGZ + SIN groups showed an overall shift toward the Sham group, with the high-dose group exhibiting a more pronounced trend toward normalization. These findings indicate that combined ligustrazine and sinomenine treatment partially restored the CCI-induced alterations in the neurotransmitter metabolic profile.

OPLS-DA was subsequently performed to further evaluate between-group differences using a supervised multivariate approach. As shown in Figure S7B, the Sham and Model groups were clearly separated, further supporting the presence of CCI-induced remodeling of neurotransmitter metabolism in the spinal dorsal horn. Compared with the Model group, the low- and medium-dose groups exhibited partial shifts, although the separation remained relatively limited. In contrast, the high-dose group showed a more distinct separation from the Model group, suggesting that high-dose combined treatment may exert broader effects on the overall neurotransmitter metabolic state. Permutation testing demonstrated clear differentiation between the original and permuted model parameters, supporting the stability and discriminatory performance of the OPLS-DA models (Figure S7C).

Based on the global metabolic profile analysis, differential metabolites were identified according to between-group statistical comparisons and subsequently subjected to KEGG pathway enrichment analysis. As shown in Figure 6A, the differential metabolites mapped to multiple pathways related to amino acid metabolism and neurotransmitter metabolism. Among these, tryptophan metabolism and tyrosine metabolism showed relatively high enrichment significance and contained comparatively large numbers of differential metabolites, indicating that CCI-induced neurotransmitter metabolic abnormalities in the spinal dorsal horn prominently involved serotonergic, kynurenine, and catecholaminergic metabolic processes.

The differential metabolites were also associated with alanine, aspartate and glutamate metabolism; D-amino acid metabolism; arginine biosynthesis; arginine and proline metabolism; glutathione metabolism; butanoate metabolism; histidine metabolism; and purine and pyrimidine metabolism. These findings indicate that CCI-induced disruption of neurotransmitter homeostasis in the spinal dorsal horn is not confined to a single neurotransmitter system but instead involves coordinated alterations in monoaminergic neurotransmission, excitatory amino acid metabolism, and related metabolic networks.

Based on the multivariate statistical analyses, differential metabolite screening, and KEGG enrichment results, subsequent analyses focused on quantitative assessment of key metabolites within the glutamine–glutamate, tryptophan–serotonin/kynurenine, and tyrosine–catecholamine metabolic axes to further characterize the specific metabolic alterations induced by CCI and modulated by combined ligustrazine and sinomenine treatment.

#### 3.5.2. Combined Ligustrazine and Sinomenine Treatment Ameliorates Glutamate Metabolic Imbalance and Modulates Abnormal GRIN2B/NR2B Expression

Among the indicators associated with the glutamine–glutamate metabolic axis, Gln showed only minor variation across groups, with no significant between-group differences (Figure 6B). In contrast, Glu levels were significantly elevated in the Model group compared with the Sham group, indicating that CCI induced abnormalities in excitatory amino acid metabolism in the spinal dorsal horn and may enhance glutamatergic neurotransmission.

Following combined ligustrazine and sinomenine treatment, Glu levels were significantly reduced in the high-dose group compared with the Model group. Glu levels also tended to decrease in the low- and medium-dose groups, although these changes did not reach statistical significance. These results indicate that CCI induces abnormal accumulation of Glu in the spinal dorsal horn, whereas combined ligustrazine and sinomenine treatment, particularly at the high dose, partially corrects this alteration.

To further investigate the relationship between altered glutamate metabolism and NMDA receptor-associated excitatory neurotransmission, the mRNA expression of GRIN2B and the protein expression of its encoded NR2B subunit were examined. GRIN2B encodes the NR2B subunit of the NMDA receptor and is an important molecule involved in glutamatergic synaptic transmission and central sensitization.

qRT-PCR analysis showed that GRIN2B mRNA expression in the spinal dorsal horn was decreased in the Model group compared with the Sham group (Figure 6E), indicating altered transcriptional regulation of GRIN2B under CCI conditions. Following combined ligustrazine and sinomenine treatment, GRIN2B mRNA expression changed to varying degrees, suggesting that the combination may modulate its transcriptional regulation.

Western blot analysis further showed that NR2B protein expression was significantly increased in the Model group compared with the Sham group (Figure 6F), indicating aberrant expression of the NMDA receptor NR2B subunit in the spinal dorsal horn following CCI. Compared with the Model group, combined ligustrazine and sinomenine treatment reduced NR2B protein expression to varying degrees, suggesting that the treatment attenuated the CCI-induced elevation of NR2B protein.

Notably, GRIN2B mRNA expression was decreased, whereas NR2B protein expression was significantly increased in the Model group, indicating discordant changes at the transcriptional and protein levels. This discrepancy suggests that abnormal NR2B expression under CCI conditions may not be governed solely by transcriptional regulation but may also involve altered mRNA translation efficiency, protein degradation, protein stability, receptor trafficking to the membrane, or other post-transcriptional regulatory mechanisms.

When considered together with the neurotransmitter data, the marked elevation of Glu accompanied by abnormal NR2B protein expression in the Model group suggests that CCI may contribute to the maintenance of central sensitization in the spinal dorsal horn by enhancing glutamatergic excitatory transmission and NMDA receptor-associated signaling. Following combined ligustrazine and sinomenine treatment, both Glu levels and NR2B protein expression were restored to varying degrees, suggesting that the combination may attenuate CCI-induced central sensitization by ameliorating glutamate metabolic imbalance and suppressing NR2B-associated excitatory neurotransmission.

Collectively, abnormalities in the glutamate metabolic axis of the spinal dorsal horn in CCI rats were characterized by elevated Glu levels, discordant GRIN2B mRNA and NR2B protein expression, and abnormal upregulation of NR2B protein. Combined ligustrazine and sinomenine treatment reduced the abnormally elevated levels of Glu and NR2B protein, suggesting that modulation of glutamate–NMDA receptor signaling may contribute to restoration of excitatory neurotransmission homeostasis in the spinal dorsal horn and thereby attenuate central sensitization during the maintenance phase of neuropathic pain.

#### 3.5.3. Combined Ligustrazine and Sinomenine Treatment Modulates the Tryptophan–Serotonin/Kynurenine Metabolic Axis

KEGG enrichment analysis identified tryptophan metabolism as one of the pathways with relatively high enrichment of differential metabolites. Quantitative analysis of metabolites associated with this pathway further showed that CCI induced broad alterations in serotonin-, kynurenine-, and indole-related metabolites downstream of tryptophan.

As shown in Figure 6C, compared with the Sham group, the Model group exhibited significantly decreased levels of 5-HTP, 5-HT, 5-HTOL, Kyn, and TrpA in the spinal dorsal horn, with Kyn showing a particularly pronounced reduction. These findings indicate that CCI affects not only serotonin itself but also its precursor, downstream metabolites, and metabolic processes involving the conversion of tryptophan toward the kynurenine and indole branches.

Following combined ligustrazine and sinomenine treatment, 5-HT levels were significantly increased in the low-, medium-, and high-dose groups compared with the Model group, indicating a consistent restoration of the CCI-induced reduction in spinal dorsal horn 5-HT. In addition, 5-HTP levels were significantly increased in the high-dose group, suggesting that high-dose treatment also promoted recovery of the serotonin precursor pool.

Within the kynurenine branch, Kyn levels were significantly increased in the high-dose group compared with the Model group, whereas the low- and medium-dose groups showed nonsignificant trends toward recovery. Levels of 5-HTOL and TrpA also showed varying degrees of restoration in some treatment groups, although the differences relative to the Model group were not statistically significant. PA showed some variation among the Model and treatment groups but did not exhibit significant between-group differences.

Collectively, CCI resulted in an overall suppression of the tryptophan metabolic axis in the spinal dorsal horn, characterized primarily by decreases in 5-HTP, 5-HT, 5-HTOL, Kyn, and TrpA. Combined ligustrazine and sinomenine treatment exerted selective regulatory effects on this metabolic axis. Restoration of 5-HT was consistently observed across all three dose groups, whereas significant improvements in 5-HTP and Kyn were predominantly observed in the high-dose group, suggesting that high-dose treatment exerted broader regulatory effects on the tryptophan metabolic network.

#### 3.5.4. Combined Ligustrazine and Sinomenine Treatment Modulates the Phenylalanine–Tyrosine–Catecholamine Metabolic Axis

KEGG enrichment analysis also identified tyrosine metabolism as one of the pathways with relatively high enrichment of differential metabolites. To further characterize the effects of CCI and combined treatment on catecholamine neurotransmitter metabolism, the levels of dopamine (DA), 3-methoxytyramine (3-MT), homovanillic acid (HVA), and norepinephrine (NE) were quantified.

As shown in Figure 6D, compared with the Sham group, the Model group exhibited significantly decreased levels of DA and NE, whereas the dopamine metabolites 3-MT and HVA were significantly increased. These findings indicate that CCI is associated with reduced levels of monoaminergic neurotransmitters such as DA and NE in the spinal dorsal horn, accompanied by abnormal accumulation of downstream dopamine metabolites, reflecting disruption of the balance among catecholamine synthesis, release, utilization, and degradation.

Following combined ligustrazine and sinomenine treatment, 3-MT levels were significantly decreased in the low-, medium-, and high-dose groups compared with the Model group. HVA levels were likewise significantly reduced across all three dose groups, indicating that the combination consistently attenuated the CCI-induced elevation of downstream dopamine metabolites.

In contrast, although DA and NE levels showed some variation following treatment, neither was significantly restored compared with the Model group. These findings suggest that the regulatory effects of combined ligustrazine and sinomenine treatment on the catecholamine metabolic axis were primarily reflected by reductions in the abnormally elevated downstream metabolites 3-MT and HVA, whereas recovery of the reduced DA and NE pools was comparatively limited.

Collectively, CCI induced catecholamine metabolic imbalance in the spinal dorsal horn, characterized by decreased DA and NE levels together with increased 3-MT and HVA levels. Combined ligustrazine and sinomenine treatment significantly reduced the abnormally elevated levels of 3-MT and HVA but did not significantly restore DA or NE, suggesting that its effects on CCI-associated catecholamine metabolic abnormalities may primarily involve modulation of dopamine turnover and downstream metabolic processes.

#### 3.5.5. Combined Ligustrazine and Sinomenine Treatment Improves Monoamine Neurotransmitter Turnover and Kynurenine Pathway Homeostasis

To further evaluate the effects of CCI and combined ligustrazine and sinomenine treatment on neurotransmitter metabolic activity and homeostatic balance in the spinal dorsal horn, metabolite ratios were calculated in addition to individual metabolite concentrations. These ratios were used to assess metabolic turnover, excitatory/inhibitory balance, and metabolic partitioning within the kynurenine pathway.

Among the indices of monoamine neurotransmitter turnover (Figure S8A), 3-MT/DA, (DOPAC + HVA)/DA, HVA/DA, Mel/5-HT, and NAS/5-HT were all significantly increased in the Model group compared with the Sham group, indicating enhanced and dysregulated monoamine neurotransmitter turnover following CCI. The pronounced increases in 3-MT/DA, (DOPAC + HVA)/DA, and HVA/DA suggest that enhanced dopamine turnover represents an important component of the monoaminergic abnormalities induced by CCI.

Following combined ligustrazine and sinomenine treatment, 3-MT/DA was significantly decreased in all three dose groups compared with the Model group. Similarly, (DOPAC + HVA)/DA was significantly reduced in the low-, medium-, and high-dose groups, indicating that the combination markedly attenuated the CCI-induced increase in dopamine turnover. HVA/DA was significantly reduced in the high-dose group, whereas the low- and medium-dose groups showed nonsignificant downward trends. NAS/5-HT was also significantly decreased in the high-dose group, while only modest reductions were observed in the low- and medium-dose groups. Although Mel/5-HT tended to decrease across the treatment groups compared with the Model group, the differences were not statistically significant. NM/NE showed only minor variation among groups and no significant differences. These findings indicate that combined ligustrazine and sinomenine treatment exerted particularly clear effects on 3-MT/DA and (DOPAC + HVA)/DA (Figure S8A).

Analysis of indices related to excitatory/inhibitory balance showed that the Gln/Glu ratio was significantly decreased in the Model group compared with the Sham group (Figure S8B), indicating disruption of glutamine–glutamate metabolic balance in the spinal dorsal horn following CCI. Combined ligustrazine and sinomenine treatment significantly increased the Gln/Glu ratio in all three dose groups compared with the Model group, indicating partial restoration of the CCI-induced imbalance in glutamine–glutamate metabolism. In contrast, the Glu/GABA ratio showed an increasing trend in the Model group relative to the Sham group, suggesting a potential shift toward excitatory neurotransmission, although no statistically significant difference was observed among groups. Following treatment, Glu/GABA tended to decrease in the low- and medium-dose groups, whereas a modest fluctuation was observed in the high-dose group. These findings indicate that CCI disrupted glutamine–glutamate metabolic homeostasis in the spinal dorsal horn, whereas combined ligustrazine and sinomenine treatment produced a clear restorative effect on the reduced Gln/Glu ratio. In contrast, changes in Glu/GABA primarily reflected a trend toward improvement rather than a statistically significant restoration of excitatory/inhibitory balance.

Analysis of metabolic balance within the kynurenine pathway showed that QA/Kyn was significantly increased, whereas Kyn/Trp was significantly decreased in the Model group compared with the Sham group (Figure S8C). These changes indicate disruption of kynurenine pathway homeostasis following CCI, characterized by reduced relative conversion of tryptophan toward the kynurenine branch together with a shift toward downstream metabolites associated with neuroexcitatory or neurotoxic activity. Following combined ligustrazine and sinomenine treatment, QA/Kyn was significantly reduced in the medium- and high-dose groups compared with the Model group, with a more pronounced reduction in the high-dose group. Kyn/Trp was significantly increased in the high-dose group, whereas the low- and medium-dose groups showed nonsignificant trends toward recovery. In contrast, KA/Kyn and KA/QA showed some variation among groups but no statistically significant differences. These findings indicate that combined ligustrazine and sinomenine treatment partially restored CCI-induced kynurenine pathway imbalance, with the most prominent effects observed for QA/Kyn in the medium- and high-dose groups and for Kyn/Trp in the high-dose group.

Overall, targeted neurotransmitter analysis demonstrated that CCI induced broad disturbances in neurotransmitter metabolism in the spinal dorsal horn, characterized by reduced serotonin- and kynurenine-related metabolites, decreased dopamine and norepinephrine levels, abnormal elevation of downstream dopamine metabolites, increased glutamate levels, enhanced monoamine neurotransmitter turnover, and disruption of kynurenine pathway homeostasis. Combined ligustrazine and sinomenine treatment restored these abnormalities to varying degrees. The high-dose group showed the most pronounced overall improvements in the neurotransmitter metabolic profile, including 5-HT- and Kyn-related metabolites, HVA and 3-MT, Glu, Gln/Glu, and QA/Kyn, suggesting that the combination can ameliorate CCI-induced disruption of neurotransmitter homeostasis in the spinal dorsal horn.

### 3.6. Combined Ligustrazine and Sinomenine Treatment Modulates Arginine–Polyamine Metabolism and Redox Homeostasis in the Spinal Dorsal Horn

#### 3.6.1. Combined Ligustrazine and Sinomenine Treatment Ameliorates Arginine–Polyamine Metabolic Disturbances and Modulates SAT1 Expression

To further characterize the regulatory effects of combined ligustrazine and sinomenine treatment on arginine metabolic abnormalities in the spinal dorsal horn of CCI rats, key metabolites within the polyamine, creatine, agmatine, and citrulline branches of arginine metabolism were subjected to targeted analysis. In parallel, the mRNA and protein expression levels of SAT1, a key regulatory enzyme in polyamine metabolism, were examined by qRT-PCR and Western blotting.

Within the polyamine metabolic branch (Figure 7A), arginine levels showed no marked differences among the groups. Compared with the Sham group, L-ornithine levels were significantly increased in the Model group, suggesting enhanced metabolic flux from arginine toward the ornithine branch following CCI. Following LGZ + SIN treatment, L-ornithine levels were significantly reduced compared with the Model group. Spermine levels were also significantly elevated in the Model group and markedly decreased after LGZ + SIN treatment, indicating that the combination partially corrected the abnormal accumulation of spermine induced by CCI. Meanwhile, both N1-acetylspermine levels and the N1-acetylspermine/spermine ratio were significantly decreased in the Model group but significantly increased following LGZ + SIN treatment. These findings suggest that polyamine acetylation and catabolic processes were altered under CCI conditions and were partially restored by the combined treatment. In contrast, putrescine, spermidine, and urea showed some variation among groups, but no statistically significant differences were observed.

Within the creatine metabolic branch (Figure 7B), guanidinoacetate and creatine showed increasing trends in the Model group and tended to decrease following LGZ + SIN treatment, although no statistically significant differences were detected. These findings suggest that CCI may exert a modest effect on creatine-related metabolism and that the combined treatment may partially modulate this alteration.

Within the agmatine metabolic branch (Figure 7C), agmatine levels tended to increase in the Model group compared with the Sham group and to decrease following LGZ + SIN treatment; however, these differences did not reach statistical significance. This pattern suggests that the agmatine branch may respond to both CCI and pharmacological intervention, although the magnitude of change appears to be relatively limited.

Within the citrulline metabolic branch (Figure 7D), citrulline levels showed only minor variation among the groups, with no statistically significant differences, suggesting that citrulline-related metabolism was not a major site of alteration during the maintenance phase of CCI under the present experimental conditions.

To further evaluate dysregulation of polyamine metabolism, SAT1 expression was examined at both the transcriptional and protein levels. SAT1 is a key rate-limiting enzyme in polyamine catabolism and catalyzes the acetylation of spermine and spermidine. As shown in Figure 7E, SAT1 mRNA expression was increased in the Model group compared with the Sham group, indicating altered transcriptional regulation of polyamine acetylation and catabolism in the spinal dorsal horn following CCI. Following LGZ + SIN treatment, SAT1 mRNA expression changed to varying degrees, suggesting that the combination modulated CCI-associated transcriptional abnormalities in SAT1.

Western blot analysis further showed that SAT1 protein expression was significantly increased in the Model group compared with the Sham group (Figure 7F), indicating that CCI affected not only SAT1 transcription but also resulted in abnormal SAT1 protein expression. Compared with the Model group, SAT1 protein expression showed a downward trend following LGZ + SIN treatment, suggesting that modulation of aberrant SAT1 expression may contribute to the restoration of polyamine metabolic homeostasis.

Notably, although SAT1 mRNA and protein expression were elevated in the Model group, N1-acetylspermine levels and the N1-acetylspermine/spermine ratio were decreased. This discordance indicates that changes in SAT1 expression do not necessarily correspond directly to the net accumulation of acetylated polyamine metabolites. One possible explanation is that CCI induces compensatory upregulation of SAT1 while simultaneously accelerating further oxidation, export, or downstream consumption of acetylated products. Therefore, the present findings are more consistent with a complex disturbance of polyamine metabolic flux following CCI rather than a simple suppression of polyamine acetylation inferred solely from reduced N1-acetylspermine levels.

Collectively, CCI-induced abnormalities in arginine metabolism were predominantly concentrated within the ornithine–polyamine branch, as reflected by increased L-ornithine and spermine levels, decreased N1-acetylspermine levels and N1-acetylspermine/spermine ratio, and abnormal elevations in SAT1 mRNA and protein expression. Following LGZ + SIN treatment, L-ornithine and spermine levels were reduced, N1-acetylspermine and its relative proportion were restored, and abnormal SAT1 expression was partially modulated. These findings suggest that combined ligustrazine and sinomenine treatment may not simply promote or inhibit a single metabolic reaction but may instead restore polyamine metabolic homeostasis in the spinal dorsal horn by correcting disturbances in ornithine–polyamine metabolic flux and SAT1-associated regulation.

#### 3.6.2. Combined Ligustrazine and Sinomenine Treatment Ameliorates Redox Metabolic Imbalance and Modulates xCT Expression

To further evaluate the regulatory effects of combined ligustrazine and sinomenine treatment on redox homeostasis in the spinal dorsal horn of CCI rats, metabolic indicators associated with the NADP/NADPH redox system, glutathione redox system, and cystine–cysteine homeostasis were analyzed. In parallel, the mRNA and protein expression levels of xCT, the key light-chain subunit of the cystine/glutamate antiporter system xc^−^, were examined by qRT-PCR and Western blotting.

Within the NADP/NADPH redox system (Figure 8A), NADPH levels were significantly increased, whereas the NADP/NADPH ratio was significantly decreased in the Model group compared with the Sham group, indicating an altered redox state in the spinal dorsal horn following CCI. After LGZ + SIN treatment, NADPH levels were significantly reduced and the NADP/NADPH ratio was significantly increased compared with the Model group, indicating that the combination partially corrected the CCI-induced imbalance in the NADP/NADPH redox system. In contrast, although NADP levels showed some variation among groups, no statistically significant differences were observed.

Within the glutathione redox system (Figure 8B), GSSG levels were significantly increased in the Model group compared with the Sham group, consistent with enhanced oxidative stress under CCI conditions. Following LGZ + SIN treatment, GSSG levels were significantly reduced compared with the Model group. GSH levels showed no marked differences among groups. The GSH/GSSG ratio tended to decrease in the Model group and to recover following LGZ + SIN treatment, although these changes did not reach statistical significance. These findings suggest that combined ligustrazine and sinomenine treatment partially ameliorated CCI-induced disturbances in glutathione redox homeostasis.

Within the cystine–cysteine system (Figure 8C), the cystine/cysteine ratio was significantly decreased in the Model group compared with the Sham group, indicating disruption of cystine–cysteine homeostasis following CCI. LGZ + SIN treatment significantly increased this ratio relative to the Model group, suggesting partial restoration of this redox-related metabolic balance. In contrast, the individual levels of cystine and cysteine showed some variation among groups but did not differ significantly.

To further investigate the molecular basis underlying alterations in cystine–cysteine metabolism, SLC7A11/xCT expression was examined at both the transcriptional and protein levels. SLC7A11 encodes xCT, the light-chain subunit of system xc^−^, which mediates extracellular cystine uptake coupled with intracellular glutamate export and is closely associated with cysteine availability and glutathione synthesis. As shown in Figure 8D, SLC7A11 mRNA expression was decreased in the Model group compared with the Sham group, indicating altered transcriptional regulation of xCT under CCI conditions. Following LGZ + SIN treatment, SLC7A11 mRNA expression changed to varying degrees, suggesting that the combination may modulate its transcriptional regulation.

Western blot analysis showed that xCT protein expression was significantly increased in the Model group compared with the Sham group (Figure 8E), indicating abnormal accumulation of xCT protein in the spinal dorsal horn following CCI. Compared with the Model group, xCT protein expression was reduced to varying degrees in the low-, medium-, and high-dose LGZ + SIN groups, with a progressively greater reduction as the treatment dose increased. These findings suggest a dose-related regulatory effect of the combination treatment on CCI-induced abnormal xCT protein expression.

Notably, SLC7A11 mRNA expression was decreased, whereas xCT protein expression was significantly increased in the Model group, indicating discordant changes at the transcriptional and protein levels. This discrepancy suggests that regulation of xCT under CCI conditions may involve not only transcriptional mechanisms but also post-transcriptional processes, including altered mRNA translation efficiency, protein degradation, protein stability, or stress-related compensatory responses. Therefore, the increase in xCT protein should not be interpreted simply as transcriptional activation of SLC7A11 but may instead reflect a complex adaptive response to CCI-induced oxidative stress. Together with the elevated GSSG level, decreased NADP/NADPH ratio, and reduced cystine/cysteine ratio observed in the Model group, these findings indicate that overall redox homeostasis in the spinal dorsal horn remained disrupted despite increased xCT protein expression.

Collectively, CCI induced pronounced disturbances in redox metabolism in the spinal dorsal horn, characterized by increased NADPH and GSSG levels, a decreased NADP/NADPH ratio, and a reduced cystine/cysteine ratio, together with discordant SLC7A11 mRNA and xCT protein expression. Following LGZ + SIN treatment, these abnormal metabolic indices were restored to varying degrees, accompanied by attenuation of the abnormal increase in xCT protein expression. These findings suggest that combined ligustrazine and sinomenine treatment may promote restoration of redox homeostasis in the spinal dorsal horn of CCI rats by improving NADP/NADPH and glutathione redox balance, modulating cystine–cysteine metabolism, and correcting abnormal xCT expression.

## 4. Discussion

The CCI model recapitulates a persistent pain state following peripheral nerve injury and is one of the classical experimental models used to investigate the mechanisms of neuropathic pain and evaluate pharmacological interventions. Following peripheral nerve injury, local inflammatory mediator release and persistent aberrant nociceptive input are transmitted to the spinal dorsal horn, where they can enhance neuronal excitability, activate glial cells, and alter synaptic plasticity, ultimately contributing to the development of central sensitization [2,20]. In the present study, rats in the Model group exhibited pronounced mechanical and cold hypersensitivity, together with reduced ipsilateral hind-paw contact area and weight bearing and prolonged swing duration. These findings indicate that CCI induced not only sensory hypersensitivity but also pain-avoidance-related motor dysfunction. Combined ligustrazine and sinomenine treatment improved these behavioral and gait abnormalities to varying degrees, with the high-dose group showing the most pronounced effects. These findings are consistent with our previous observations [14] and further support the reproducible antinociceptive and functional benefits of this combination in CCI-induced neuropathic pain.

Importantly, the persistence of neuropathic pain is closely associated with remodeling of the local metabolic microenvironment in the spinal dorsal horn. Integrated transcriptomic and widely targeted metabolomic analyses in the present study showed that CCI induced substantial alterations in both gene expression and metabolic profiles, whereas combined ligustrazine and sinomenine treatment partially normalized subsets of these abnormal genes and metabolites. Joint pathway analysis further highlighted amino acid-related pathways, particularly arginine and proline metabolism and pathways related to phenylalanine, tyrosine, and tryptophan metabolism, as potential points of convergence between CCI pathology and pharmacological intervention. These findings are biologically plausible because arginine metabolism is closely linked to polyamine production, nitric oxide synthesis, the urea cycle, and redox regulation, whereas aromatic amino acid metabolism is directly connected to serotonin, catecholamine, and kynurenine pathways. Together, these pathways provide an interface among inflammatory signaling, neurotransmitter metabolism, oxidative stress, and central sensitization. Thus, the effects of combined ligustrazine and sinomenine treatment are unlikely to be attributable to modulation of a single target but may instead reflect broader remodeling of metabolic networks within the spinal dorsal horn.

Oxylipins are bioactive lipid mediators generated from polyunsaturated fatty acids through cyclooxygenase, lipoxygenase, and cytochrome P450 pathways and participate in inflammation, vascular regulation, immune-cell function, and the resolution of inflammation [21]. In the present study, targeted oxylipin profiling revealed that CCI disrupted DHA-, EPA-, AA-, and LA/ALA-related metabolic pathways in the spinal dorsal horn, indicating widespread disturbance of local lipid mediator networks under neuropathic pain conditions. Specifically, the Model group showed decreased DHA and increased EPA levels, together with abnormal elevations in the DHA-derived metabolite (±)-20-HDoHE and the EPA-derived metabolite 19(20)-EpDPE, suggesting altered ω-3 PUFA metabolism and downstream oxylipin formation. Combined ligustrazine and sinomenine treatment reduced the abnormally elevated levels of these key oxylipins and shifted DHA/EPA-related metabolic patterns toward those observed in the Sham group. These findings suggest that modulation of lipid mediator metabolism may contribute to improvement of the inflammatory and metabolic microenvironment within the spinal dorsal horn.

Disruption of neurotransmitter homeostasis is another important feature associated with central sensitization in the spinal dorsal horn. Persistent peripheral nociceptive input can alter monoaminergic neurotransmission, excitatory amino acid signaling, and tryptophan–kynurenine metabolism, thereby affecting descending pain modulation, synaptic transmission, and neuroimmune interactions [22]. In the present study, CCI reduced the levels of 5-HTP, 5-HT, 5-HTOL, Kyn, and TrpA in the spinal dorsal horn, indicating suppression of the tryptophan–serotonin/kynurenine/indole metabolic axis. The reduction in 5-HT may compromise descending inhibitory pain modulation at the spinal level, whereas decreases in Kyn and the Kyn/Trp ratio suggest reduced relative metabolic flux from tryptophan into the kynurenine pathway. Combined ligustrazine and sinomenine treatment significantly restored 5-HT across all three dose groups, while the high-dose treatment also increased 5-HTP and Kyn. These findings suggest that the combination may improve neurotransmitter homeostasis in the spinal dorsal horn, at least in part, by restoring neuroactive metabolites derived from tryptophan.

Downstream metabolites of the kynurenine pathway exert complex neuroactive effects. Quinolinic acid acts as an NMDA receptor agonist, whereas kynurenic acid is generally considered to exert neuroprotective and NMDA receptor-antagonistic effects [23,24]. In the present study, QA/Kyn was increased and Kyn/Trp was decreased in the Model group, suggesting that CCI not only reduced the relative entry of tryptophan into the kynurenine pathway but may also have shifted downstream metabolism toward a more neuroexcitatory or neurotoxic profile. Medium- and high-dose combined ligustrazine and sinomenine treatment reduced QA/Kyn, while the high-dose treatment also increased Kyn/Trp. These findings indicate that the combination partially restores metabolic balance within the kynurenine pathway and provide additional metabolic evidence for its ability to ameliorate the abnormal central state associated with CCI.

Within the phenylalanine–tyrosine–catecholamine metabolic axis, CCI reduced DA and NE levels while increasing HVA and 3-MT, accompanied by elevations in dopamine turnover indices, including 3-MT/DA, (DOPAC + HVA)/DA, and HVA/DA. DA and NE are important monoaminergic neurotransmitters involved in descending pain modulation, and dysregulation of these systems has been closely associated with chronic pain processing [25]. Our findings therefore suggest that CCI may produce a state in which reduced monoamine availability coexists with enhanced dopamine turnover in the spinal dorsal horn. Combined ligustrazine and sinomenine treatment significantly reduced HVA, 3-MT, and several dopamine turnover ratios, indicating partial correction of the CCI-induced catecholamine metabolic disturbance. Although DA and NE themselves showed only trends toward recovery without reaching statistical significance, the normalization of their downstream metabolites and turnover indices suggests that stabilization of monoamine metabolic activity may contribute to the analgesic effects of the combination.

Glutamate is the principal excitatory neurotransmitter involved in synaptic transmission in the spinal dorsal horn, and excessive glutamatergic signaling can enhance NMDA receptor-mediated synaptic plasticity, a central mechanism underlying central sensitization [26]. In the present study, Glu levels were elevated and the Gln/Glu ratio was reduced in the spinal dorsal horn of CCI rats, indicating disruption of glutamine–glutamate cycling and excitatory amino acid homeostasis. High-dose combined ligustrazine and sinomenine treatment significantly reduced Glu levels, whereas all dose groups increased the Gln/Glu ratio, suggesting restoration of glutamine–glutamate metabolic balance. When considered together with the observed changes in NR2B protein expression, these findings suggest that the combination may attenuate central sensitization by reducing glutamate-associated excitatory pressure and modulating NMDA receptor-related signaling.

Arginine–polyamine metabolism emerged as another key abnormal pathway from the integrated omics analysis. CCI altered L-ornithine, spermine, N1-acetylspermine, and the N1-acetylspermine/spermine ratio, indicating disruption of the metabolic branch through which arginine is directed via ornithine into polyamine metabolism. N1-Acetylspermine and its ratio to spermine can, to some extent, reflect the balance of spermine acetylation and catabolic turnover, while SAT1 is a major regulatory enzyme in this process. Combined ligustrazine and sinomenine treatment restored N1-acetylspermine and the N1-acetylspermine/spermine ratio and modulated SAT1 expression, suggesting that it corrected CCI-associated disturbances in polyamine metabolic regulation. Given that polyamines can modulate NMDA receptors and multiple ion channels, normalization of polyamine metabolism may reduce the influence of abnormal polyamine turnover on neuronal excitability and synaptic plasticity. Accordingly, arginine–polyamine metabolism may represent an important link between metabolic remodeling and central sensitization in the spinal dorsal horn and a potential metabolic target of combined ligustrazine and sinomenine treatment.

With respect to redox metabolism, CCI produced abnormalities in the NADP/NADPH system, GSSG levels, and the cystine/cysteine ratio, indicating disturbances in reducing-equivalent availability, glutathione redox status, and sulfur-containing amino acid metabolism in the spinal dorsal horn. These abnormalities were partially corrected by combined ligustrazine and sinomenine treatment, suggesting that the combination can restore local redox homeostasis and alleviate metabolic stress associated with persistent nerve injury. Similar multidimensional regulatory effects have been reported for other naturally derived bioactive compounds, which can simultaneously modulate inflammatory responses, oxidative stress, and cellular homeostasis [27]. This supports the concept that coordinated regulation of inflammation and redox status may represent an important pharmacological feature of naturally derived bioactive agents. Notably, SLC7A11/xCT-mediated cystine–glutamate exchange links glutathione synthesis directly to extracellular glutamate regulation. In the present study, alterations in xCT expression and cystine/cysteine metabolism occurred in parallel with increased Glu and a reduced Gln/Glu ratio, suggesting possible coupling between antioxidant substrate availability and excitatory glutamatergic signaling under CCI conditions. The coordinated normalization of these indices following treatment further suggests that combined ligustrazine and sinomenine may simultaneously alleviate oxidative stress and excessive excitatory signaling by restoring the balance among cystine uptake, glutathione metabolism, and glutamate transport.

NR2B (GluN2B) is an important regulatory subunit of NMDA receptors, and alterations in its expression, trafficking, and synaptic localization can influence NMDA receptor function and synaptic plasticity in the spinal dorsal horn. Recent studies have implicated enhanced spinal dorsal horn NMDA receptor activity, particularly abnormal GluN2B-associated receptor function, in amplification of nociceptive input and maintenance of central sensitization in neuropathic pain [28,29,30]. In the present study, CCI induced abnormal GRIN2B/NR2B expression, which was modulated by combined ligustrazine and sinomenine treatment. Together with the increase in Glu, reduction in the Gln/Glu ratio, and alterations in xCT, these findings support a potential interaction among glutamate metabolic dysregulation, redox imbalance, and enhanced NMDA receptor-associated excitatory signaling in the spinal dorsal horn following CCI. Combined ligustrazine and sinomenine treatment may therefore reduce abnormal spinal excitability by simultaneously regulating glutamate metabolism, xCT-mediated cystine/glutamate exchange, and NR2B-associated signaling, thereby ameliorating pain sensitization.

In conclusion, the present study demonstrates that combined ligustrazine and sinomenine treatment ameliorates mechanical hypersensitivity, cold hypersensitivity, and gait dysfunction in CCI rats, with its effects potentially involving multiple targets and multiple regulatory pathways in modulating systemic inflammatory dysregulation and restoring metabolic homeostasis in the spinal dorsal horn. Specifically, the combination ameliorated DHA/EPA-related oxylipin metabolic disturbances, restored the tryptophan–serotonin/kynurenine and catecholamine metabolic axes, attenuated abnormalities in glutamate metabolism, modulated arginine–polyamine metabolism and redox status, and influenced xCT/NR2B-associated central sensitization. These findings provide experimental support for combined ligustrazine and sinomenine as a potential multitarget therapeutic strategy for neuropathic pain and further suggest that remodeling of the metabolic microenvironment in the spinal dorsal horn may represent an important mechanistic basis for its antinociceptive effects.

## 5. Conclusions

In conclusion, this study demonstrates that combined ligustrazine and sinomenine treatment alleviates CCI-induced neuropathic pain and central sensitization by restoring metabolic homeostasis in the spinal dorsal horn. Integrated transcriptomic and metabolomic analyses revealed that the therapeutic effects of LGZ + SIN were associated with coordinated regulation of multiple metabolic networks, including arginine–polyamine metabolism, neurotransmitter homeostasis, oxylipin metabolism, and redox balance. These metabolic alterations were accompanied by improved inflammatory profiles, reduced oxidative stress, and modulation of pain-related signaling pathways, including the xCT/NR2B-associated axis. Collectively, our findings provide new insights into the multi-dimensional mechanisms underlying the analgesic effects of combined ligustrazine and sinomenine and highlight spinal metabolic remodeling as a potential therapeutic target for neuropathic pain.

## Figures and Tables

**Figure 1:**
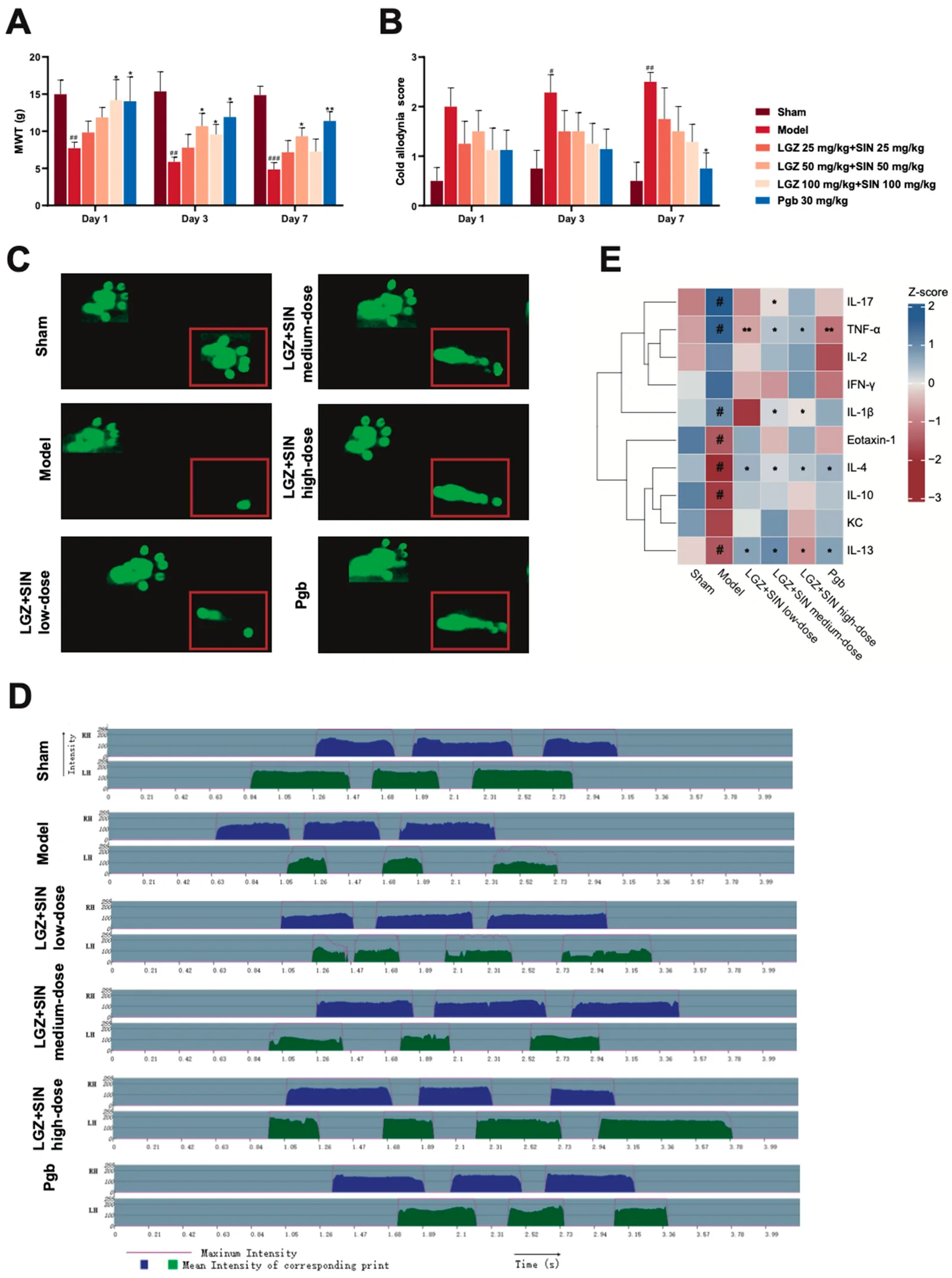
Effects of combined ligustrazine and sinomenine treatment on pain-related behaviors, gait function, and plasma inflammatory profiles in CCI rats. (**A**) Effects of 7 consecutive days of combined ligustrazine and sinomenine treatment on the mechanical withdrawal threshold (MWT) in CCI rats. (**B**) Effects of 7 consecutive days of combined ligustrazine and sinomenine treatment on cold hypersensitivity scores in CCI rats. (**C**) Representative hind-paw footprints during locomotion; the ipsilateral hind-paw footprints are indicated by red boxes. (**D**) Maximum contact intensity and pressure–time profiles of the hind paws. RH, right hind paw (contralateral side); LH, left hind paw (ipsilateral side). The purple curves indicate maximum contact intensity, whereas the blue and green shaded areas represent changes in the mean contact intensity of the corresponding paw over time. (**E**) Z-score-normalized heatmap of plasma inflammatory mediator profiles in CCI rats following combined ligustrazine and sinomenine treatment. Data are presented as the mean ± SEM. ^#^ *p* < 0.05, ^##^ *p* < 0.01 and ^###^ *p* < 0.001 vs. the Sham group; * *p* < 0.05 and ** *p* < 0.01 vs. the Model group.

**Figure 2:**
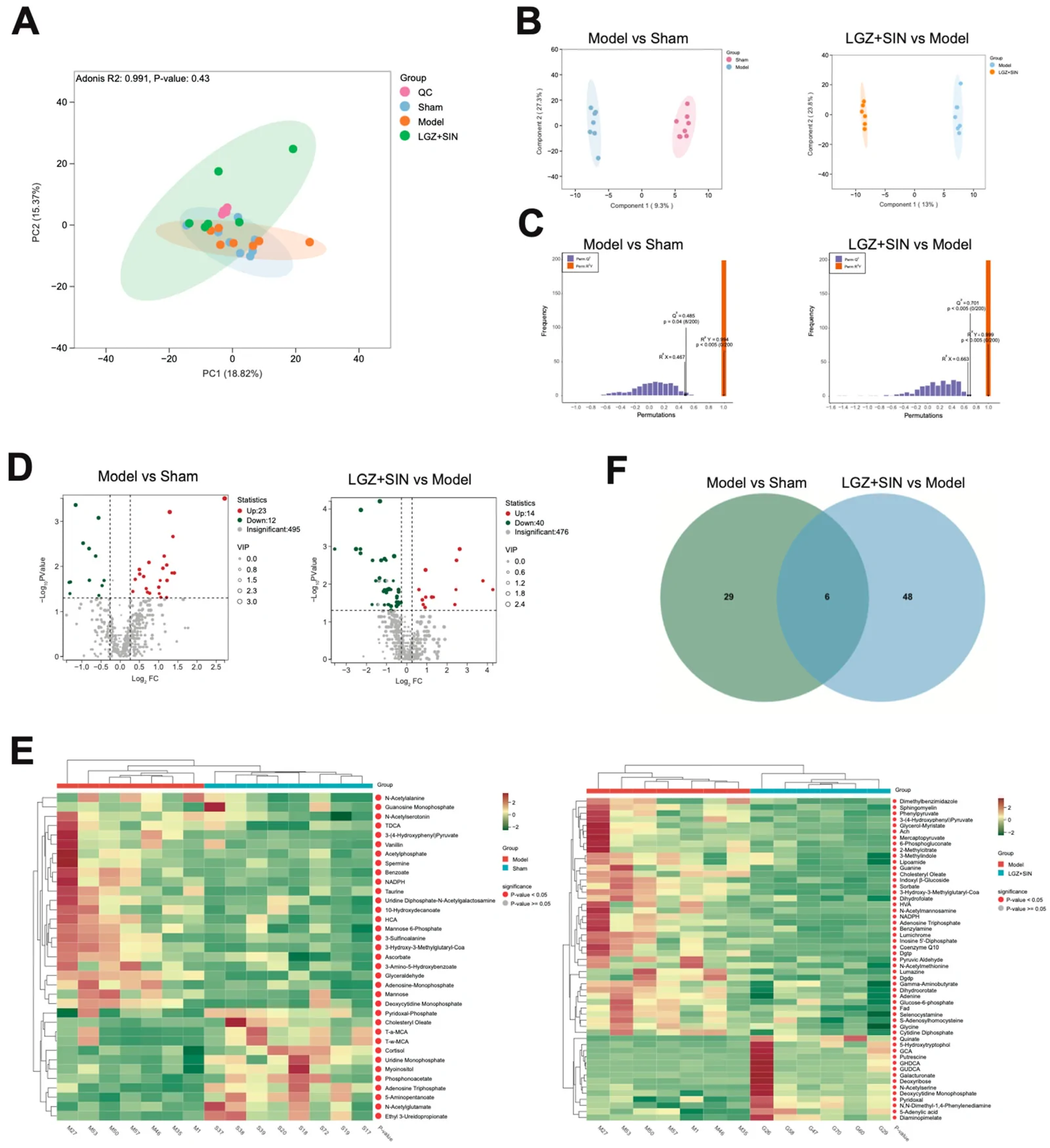
Combined ligustrazine and sinomenine treatment modulates the metabolic profile of the spinal dorsal horn in CCI rats. (**A**) Principal component analysis (PCA) score plot. (**B**) Orthogonal partial least-squares discriminant analysis (OPLS-DA) score plots for pairwise group comparisons. (**C**) Permutation test results for the OPLS-DA models. (**D**) Volcano plots of differential metabolites in the indicated group comparisons. (**E**) Hierarchical clustering heatmaps of differential metabolites. (**F**) Venn diagram showing the overlap of differential metabolites identified in the Model vs. Sham and LGZ + SIN vs. Model comparisons.

**Figure 3:**
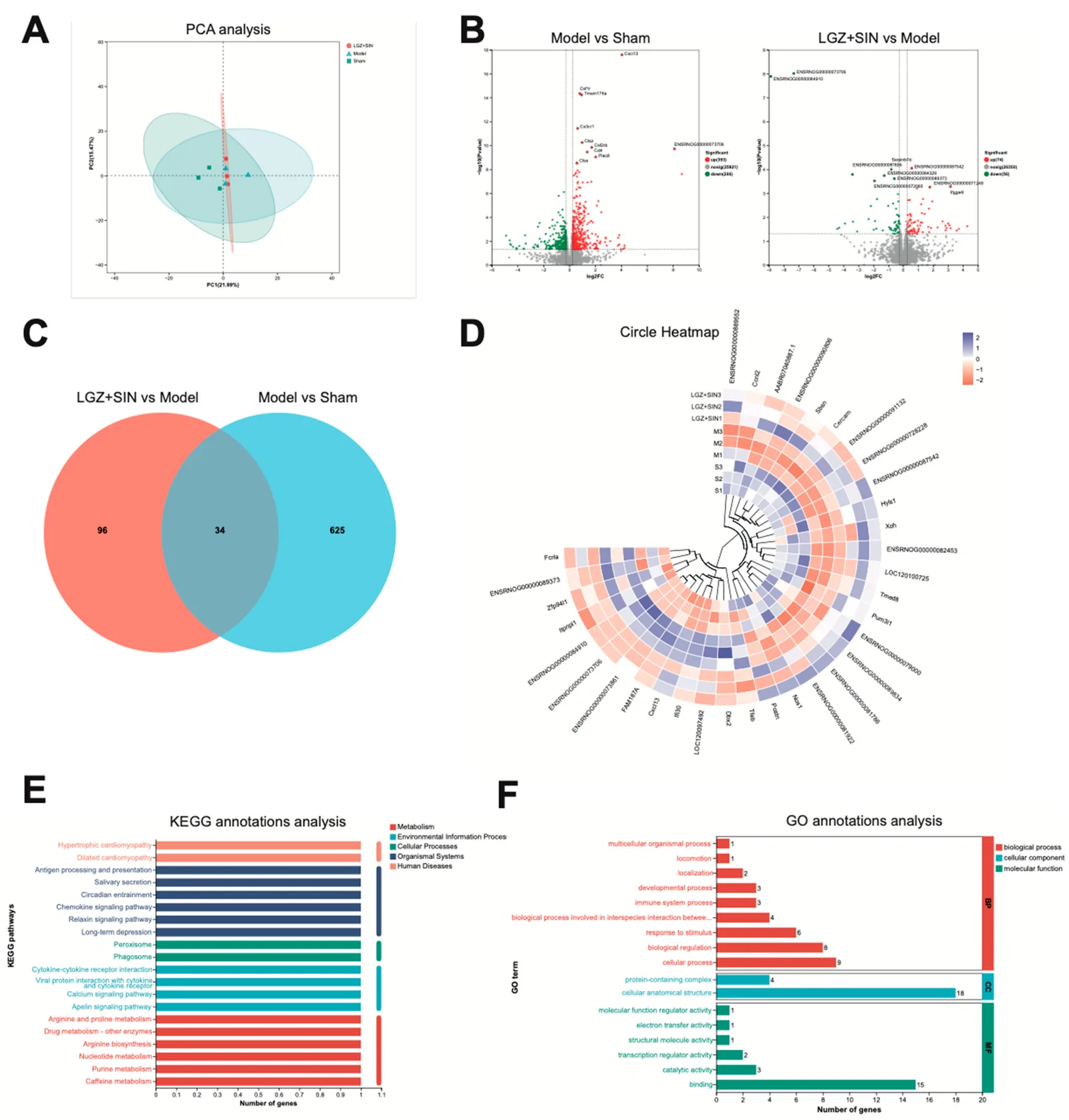
Combined ligustrazine and sinomenine treatment modulates transcriptional profiles and the functional characteristics of differentially expressed genes in the spinal dorsal horn of CCI rats. (**A**) PCA score plot of spinal dorsal horn transcriptomic samples from the indicated groups. (**B**) Volcano plots of differentially expressed genes (DEGs) for the indicated pairwise comparisons; red indicates upregulated genes, green indicates downregulated genes, and gray indicates genes without significant differential expression. (**C**) Venn diagram showing the overlap of DEGs between the two pairwise comparisons. (**D**) Circular hierarchical clustering heatmap showing the expression patterns of the 34 overlapping DEGs across the experimental groups. (**E**) KEGG pathway annotation of the overlapping DEGs. (**F**) Gene Ontology (GO) functional annotation of the overlapping DEGs.

**Figure 4:**
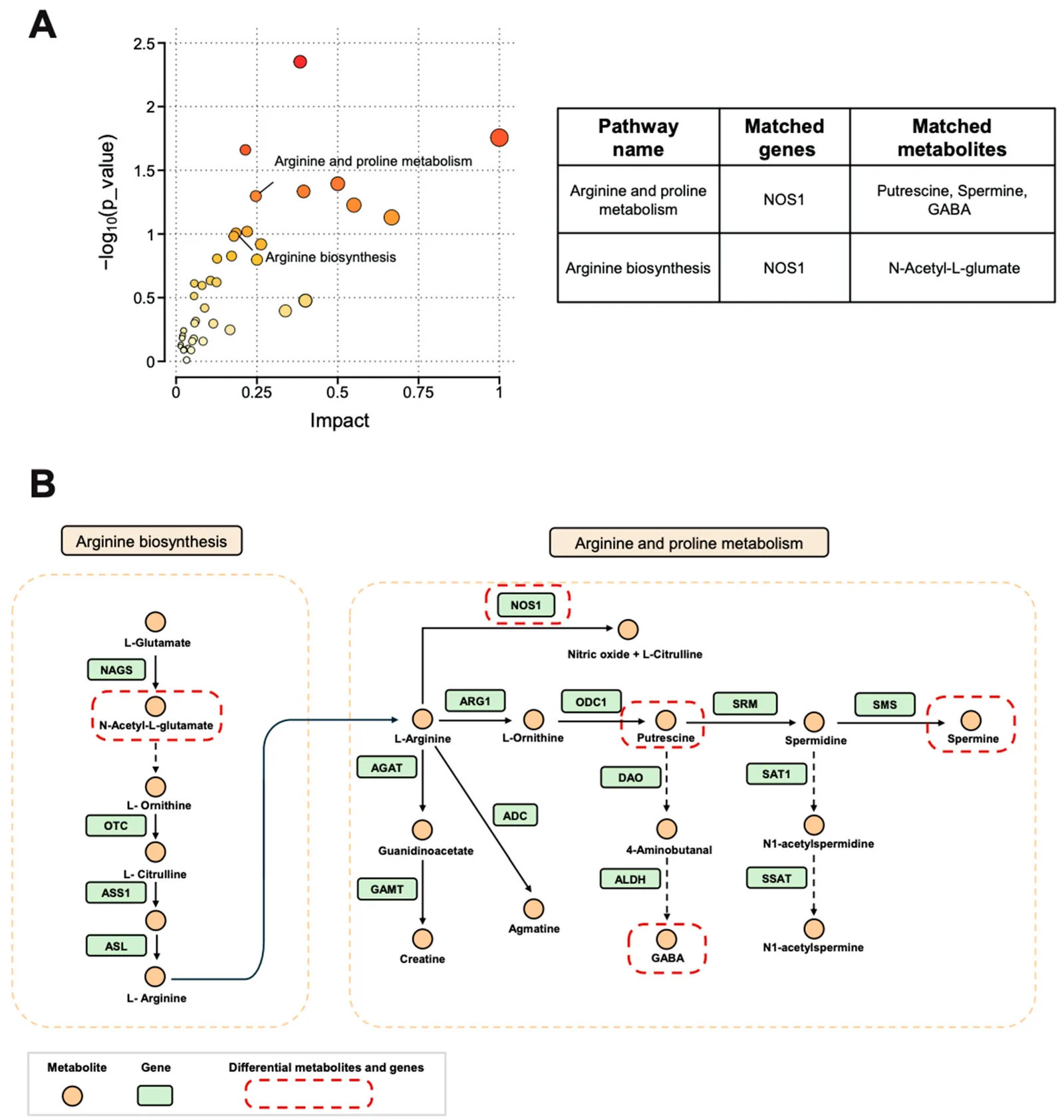
Integrated transcriptomic and metabolomic analysis reveals alterations in arginine-related metabolic networks in the spinal dorsal horn of CCI rats. (**A**) Bubble plot from the Joint Pathway Analysis of transcriptomic and metabolomic data, together with the matched molecules in arginine-related pathways. The x-axis represents pathway impact, and the y-axis represents −log_10_ (*p* value). The accompanying table lists the matched DEGs and differential metabolites in arginine and proline metabolism and arginine biosynthesis. (**B**) Locally integrated pathway map of arginine biosynthesis and arginine and proline metabolism. Orange circles represent metabolites, green rectangles represent associated genes or enzymes, and red dashed boxes indicate the differential genes or metabolites identified in the present study.

**Figure 5:**
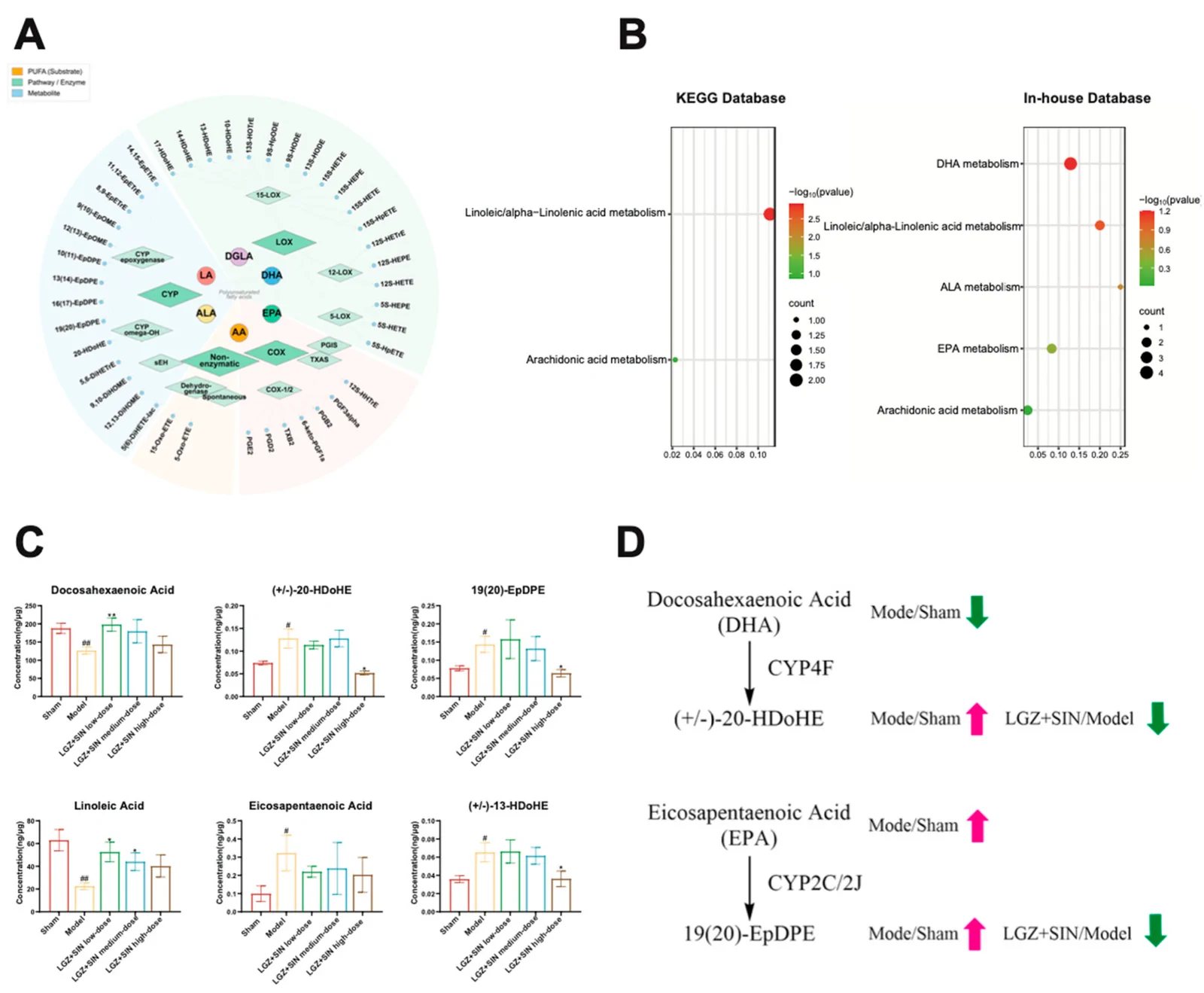
Combined ligustrazine and sinomenine treatment ameliorates oxylipin metabolic disturbances in the spinal dorsal horn of CCI rats. (**A**) Profile of the 49 quantifiable polyunsaturated fatty acids and their COX-, CYP450-, and LOX-derived metabolites detected in rat spinal dorsal horn samples. (**B**) Pathway enrichment analysis of differential oxylipins using the KEGG database and the in-house oxylipin pathway database. (**C**) Alterations in representative key oxylipins. (**D**) Schematic representation of key oxylipin metabolic pathways affected by combined ligustrazine and sinomenine treatment in CCI rats. Data are presented as the mean ± SEM. ^#^ *p* < 0.05 and ^##^ *p* < 0.01 vs. the Sham group; * *p* < 0.05 and ** *p* < 0.01 vs. the Model group.

**Figure 6:**
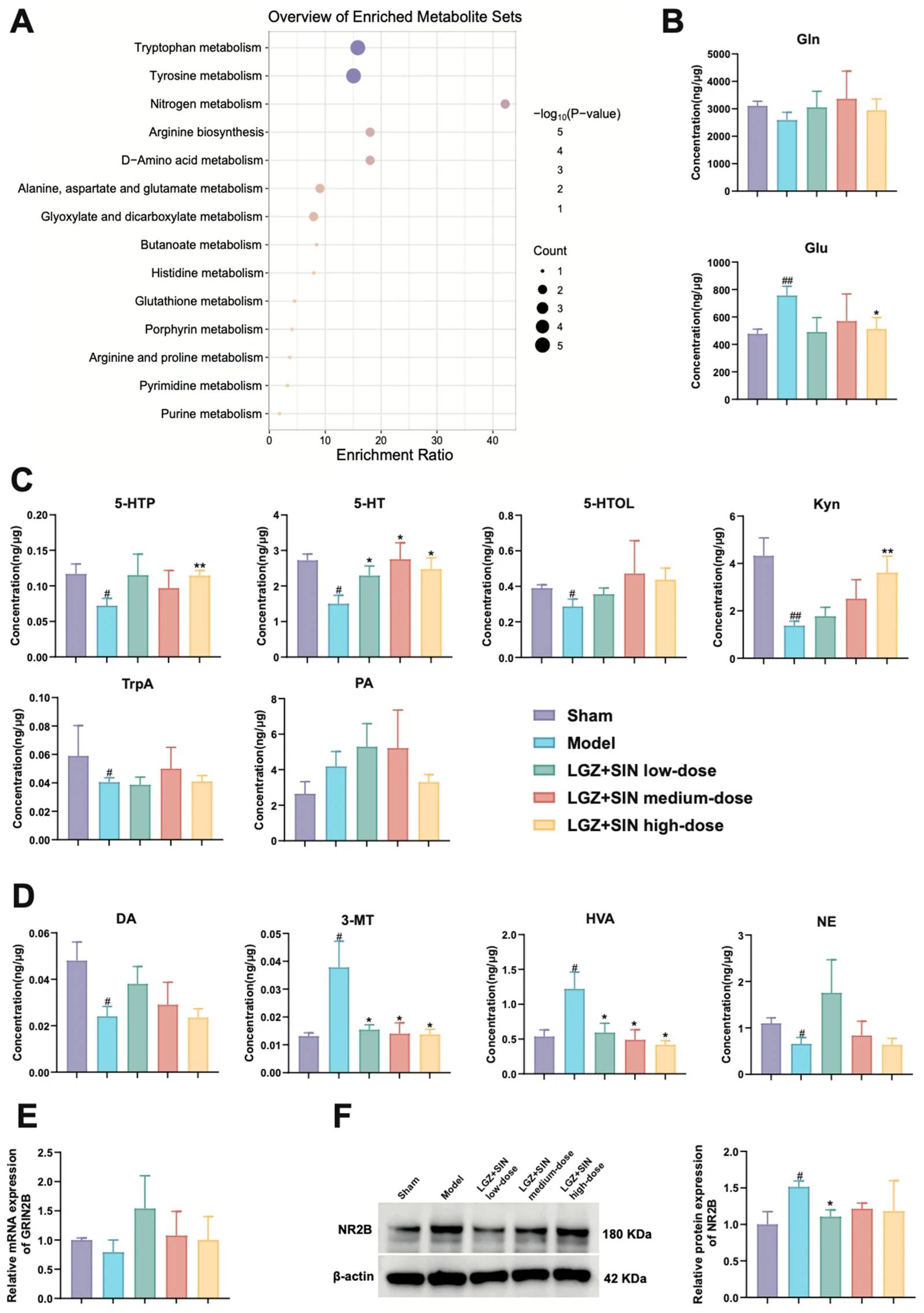
Combined ligustrazine and sinomenine treatment modulates differential neurotransmitter-related metabolic pathways and key metabolites in the spinal dorsal horn of CCI rats. (**A**) Bubble plot of KEGG pathway enrichment analysis based on differential neurotransmitter-related metabolites. (**B**) Levels of glutamine (Gln) and glutamate (Glu) associated with glutamine–glutamate metabolism. (**C**) Levels of 5-HTP, 5-HT, 5-HTOL, Kyn, TrpA, and PA associated with tryptophan–serotonin/kynurenine metabolism. (**D**) Levels of DA, 3-MT, HVA, and NE associated with tyrosine–catecholamine metabolism. (**E**) Relative mRNA expression of GRIN2B. (**F**) Representative Western blot bands and densitometric quantification of NR2B protein expression. Data are presented as the mean ± SEM. Metabolite concentrations are expressed as ng/μg protein. ^#^ *p* < 0.05 and ^##^ *p* < 0.01 vs. the Sham group; * *p* < 0.05 and ** *p* < 0.01 vs. the Model group.

**Figure 7:**
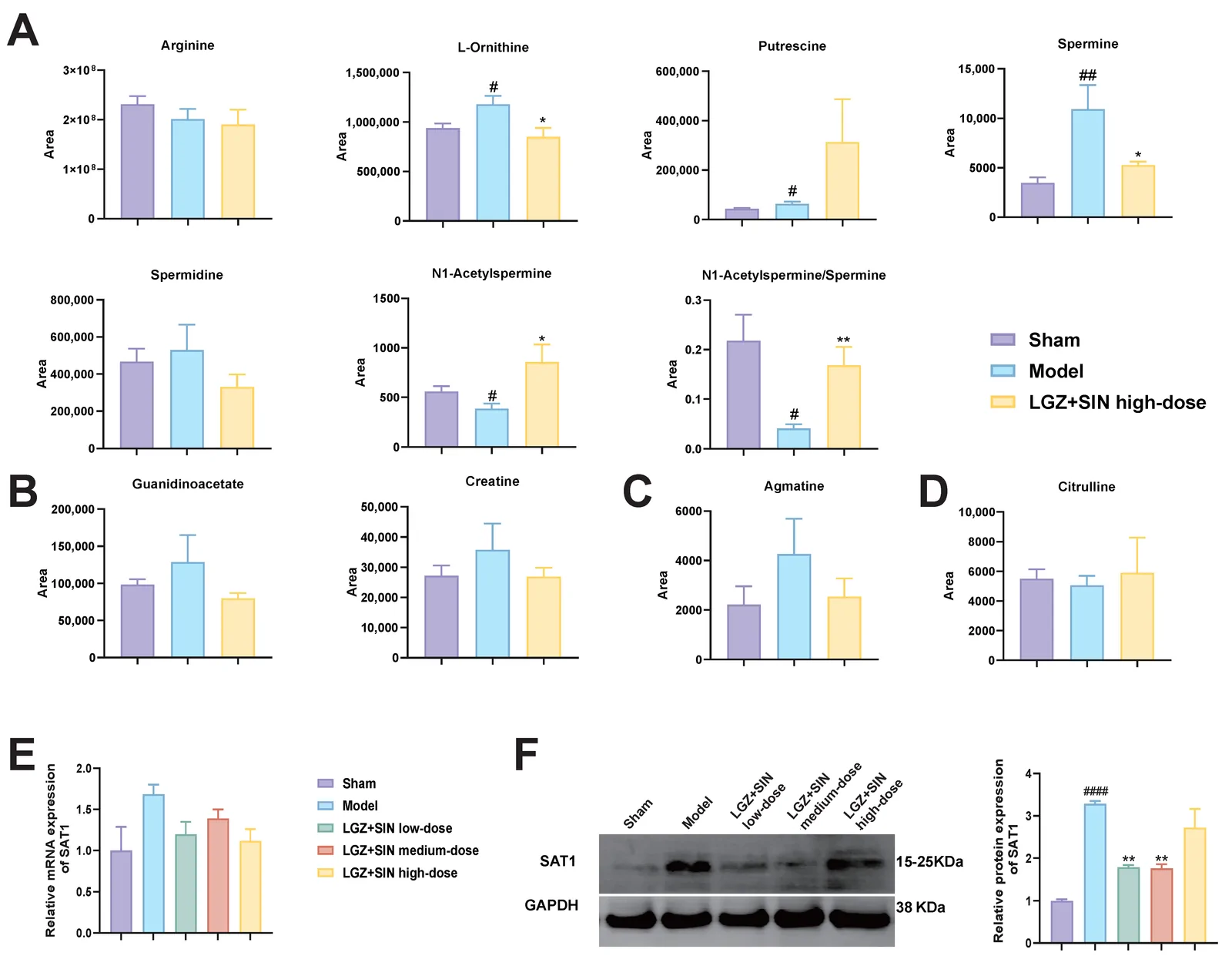
Combined ligustrazine and sinomenine treatment ameliorates arginine–polyamine metabolic disturbances and modulates SAT1 expression in CCI rats. Figure 6. Combined ligustrazine and sinomenine treatment modulates differential neurotransmitter-related metabolic pathways and key metabolites in the spinal dorsal horn of CCI rats. (**A**) Bubble plot of KEGG pathway enrichment analysis based on differential neurotransmitter-related metabolites. (**B**) Levels of glutamine (Gln) and glutamate (Glu) associated with glutamine–glutamate metabolism. (**C**) Levels of 5-HTP, 5-HT, 5-HTOL, Kyn, TrpA, and PA associated with tryptophan–serotonin/kynurenine metabolism. (**D**) Levels of DA, 3-MT, HVA, and NE associated with tyrosine–catecholamine metabolism. (**E**) Relative mRNA expression of GRIN2B. (**F**) Representative Western blot bands and densitometric quantification of NR2B protein expression. Data are presented as the mean ± SEM. Metabolite concentrations are expressed as ng/μg protein. ^#^ *p* < 0.05, ^##^ *p* < 0.01 and ^####^ *p* < 0.0001 vs. the Sham group; * *p* < 0.05 and ** *p* < 0.01 vs. the Model group.

**Figure 8:**
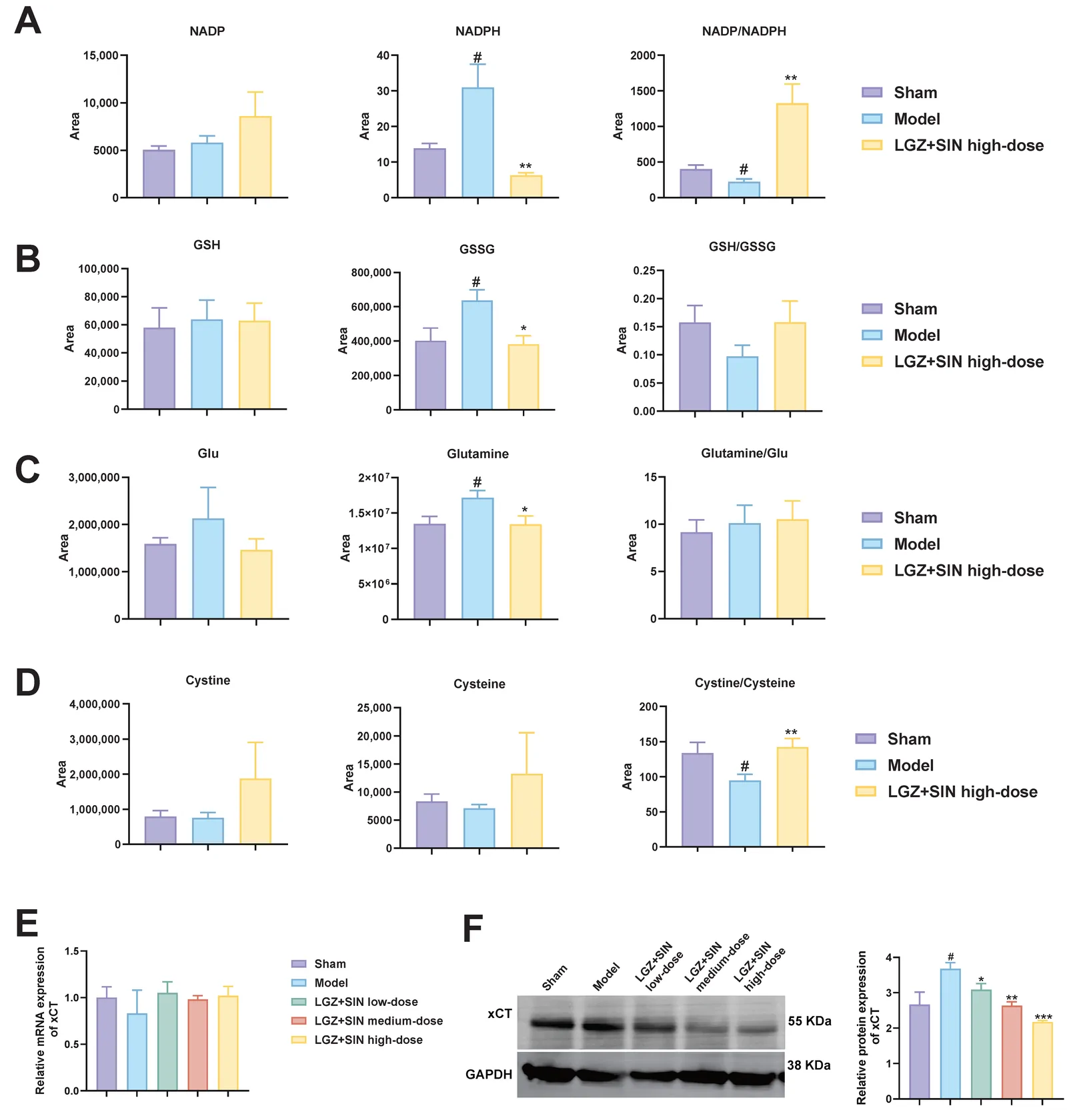
Combined ligustrazine and sinomenine treatment ameliorates redox metabolic imbalance and modulates xCT expression in the spinal dorsal horn of CCI rats. (**A**) NADP and NADPH levels and the NADP/NADPH ratio. (**B**) GSH and GSSG levels and the GSH/GSSG ratio. (**C**) Glu and glutamine levels and the glutamine/glu ratio. (**D**) Cystine and cysteine levels and the cystine/cysteine ratio. (**E**) Relative mRNA expression of SLC7A11/xCT. (**F**) Representative Western blot bands and densitometric quantification of xCT protein expression. Data are presented as the mean ± SEM. ^#^ *p* < 0.05 vs. the Sham group; * *p* < 0.05, ** *p* < 0.01 and *** *p* < 0.001 vs. the Model group.

**Table 1:** Effects of combined ligustrazine and sinomenine treatment on hind-paw gait symmetry parameters in CCI rats (mean ± SEM, n = 8).

Parameter	Sham	Model	LGZ + SIN Low-Dose	LGZ + SIN Medium-Dose	LGZ + SIN High-Dose	Pgb
Stance duration, LH/RH (%)	103.4 ± 3.0	70.2 ± 6.1 ^#^	72.7 ± 2.5	78.2 ± 4.5	88.6 ± 3.6	92.1 ± 2.6 *
Swing duration, LH/RH (%)	98.0 ± 6.4	216.7 ± 22.6 ^##^	167.7 ± 10.6	175.7 ± 13.7	151.7 ± 10.0 *	161.2 ± 5.8
Stance phase, LH/RH (%)	100.2 ± 3.3	70.3 ± 5.9 ^##^	73.4 ± 2.6	93.2 ± 2.9 *	92.8 ± 2.1 *	88.8 ± 1.8 *
Single-hind-paw support duration, LH/RH (%)	104.4 ± 6.2	64.4 ± 4.7 ^###^	68.4 ± 6.1	80.6 ± 7.8 *	81.3 ± 6.8 *	88.8 ± 2.6 *
Maximum contact area, LH/RH (%)	102.8 ± 9.0	53.1 ± 9.7 ^##^	55.3 ± 7.0	80.9 ± 3.2 *	85.5 ± 4.4 *	74.5 ± 9.7

Note: All parameters are expressed as the percentage ratio of the ipsilateral to contralateral hind paw (LH/RH). ^#^ *p* < 0.05, ^##^ *p* < 0.01, and ^###^ *p* < 0.001 versus the Sham group; * *p* < 0.05 versus the Model group.

## Data Availability

The original contributions presented in this study are included in the article/Supplementary Material. Further inquiries can be directed to the corresponding author.

## Supplementary Materials

The following supporting information can be downloaded at: https://www.mdpi.com/article/10.3390/metabo16100717/s1. Figure S1: Effects of combined ligustrazine and sinomenine treatment on body weight gain in CCI rats. Data are presented as the mean ± SEM. # *p* < 0.05, ## *p* < 0.01 and ### *p* < 0.001 vs. the Sham group; * *p* < 0.05 and ** *p* < 0.01 vs. the Model group; Figure S2: MRM chromatograms of arachidonic acid (AA) and its metabolites derived from the COX, CYP450, and LOX pathways. (A) COX-derived metabolites, comprising 39 analytes; (B) CYP450-derived metabolites, comprising 11 analytes; (C) LOX-derived metabolites, comprising 19 analytes; Figure S3: MRM chromatograms of eicosapentaenoic acid (EPA) and its metabolites derived from the COX, CYP450, and LOX pathways. (A) COX-derived metabolites, comprising 6 analytes; (B) CYP450-derived metabolites, comprising 9 analytes; (C) LOX-derived metabolites, comprising 8 analytes; Figure S4: MRM chromatograms of docosahexaenoic acid (DHA) and its metabolites derived from the CYP450 and LOX pathways. (A) CYP450-derived metabolites, comprising 10 analytes; (B) LOX-derived metabolites, comprising 19 analytes; Figure S5. MRM chromatograms of linoleic acid (LA) and its metabolites derived from the CYP450 and LOX pathways. (A) CYP450-derived metabolites, comprising 4 analytes; (B) LOX-derived metabolites, comprising 5 analytes; Figure S6. MRM chromatograms of eicosapentaenoic acid (EPA) and its metabolites derived from the COX and LOX pathways. (A) COX-derived metabolites, comprising 5 analytes; (B) LOX-derived metabolites, comprising 3 analytes; Figure S7. Multivariate statistical analysis of the neurotransmitter metabolic profiles in the spinal dorsal horn of CCI rats following combined ligustrazine and sinomenine treatment. (A) PCA score plot of the experimental groups; (B) OPLS-DA score plots for the indicated group comparisons; (C) permutation test results for the OPLS-DA models; Figure S8. Effects of combined ligustrazine and sinomenine treatment on neurotransmitter metabolic turnover and homeostatic balance in the spinal dorsal horn of CCI rats. (A) Indices of monoamine neurotransmitter turnover; (B) indices related to excitatory/inhibitory balance; (C) indices of metabolic homeostasis within the kynurenine pathway. All ratio-based indices are expressed as metabolite peak-area ratios. Data are presented as the mean ± SEM. # *p* < 0.05 and ## *p* < 0.01 vs. the Sham group; * *p* < 0.05 and ** *p* < 0.01 vs. the Model group; Table S1: qRT-PCR primer sequences; Table S2: Overlapping differential metabolites identified from Model versus Sham and LGZ + SIN versus Model comparisons; Table S3: Overlapping expressed genes identified from Model versus Sham and LGZ + SIN versus Model comparisons.
