# Supplementary material for: Ligustrazine Combined with Sinomenine Alleviates Central Sensitization in CCI-Induced Neuropathic Pain in Rats by Restoring Metabolic Homeostasis in the Spinal Dorsal Horn
Source: Metabolites. 2026 Sep 28;16(10):717. doi: 10.3390/metabo16100717 (PMC13707340; doi:10.3390/metabo16100717)
Supplement: Supplementary file 1 [file metabolites-16-00717-s001.zip › metabolites-4560973-supplementary.pdf]

Supplementary Data

Supplementary Figure S1-S8

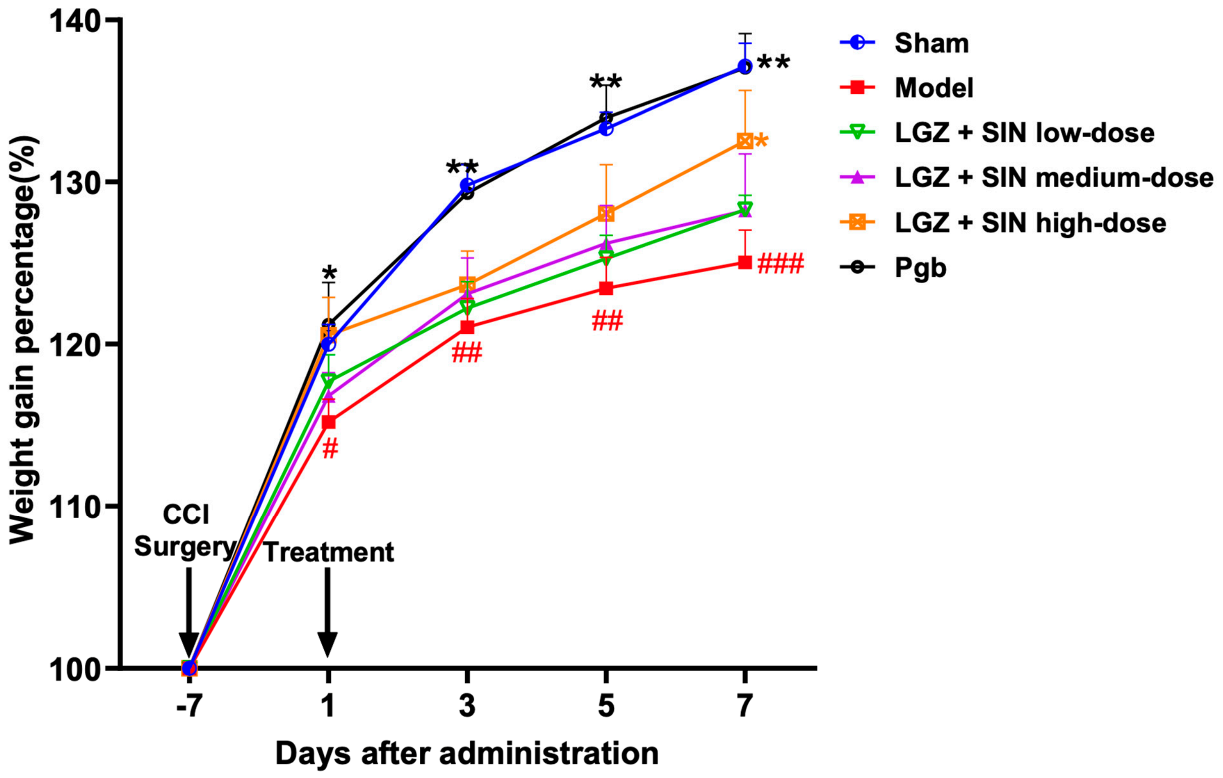

Figure S1. Effects of combined ligustrazine and sinomenine treatment on body weight gain in CCI rats. Data are presented as the mean ± SEM. $^{\#}P < 0.05$, $^{\#\#}P < 0.01$ and $^{\#\#\#}P < 0.001$ vs. the Sham group; $^{*}P < 0.05$ and $^{**}P < 0.01$ vs. the Model group.

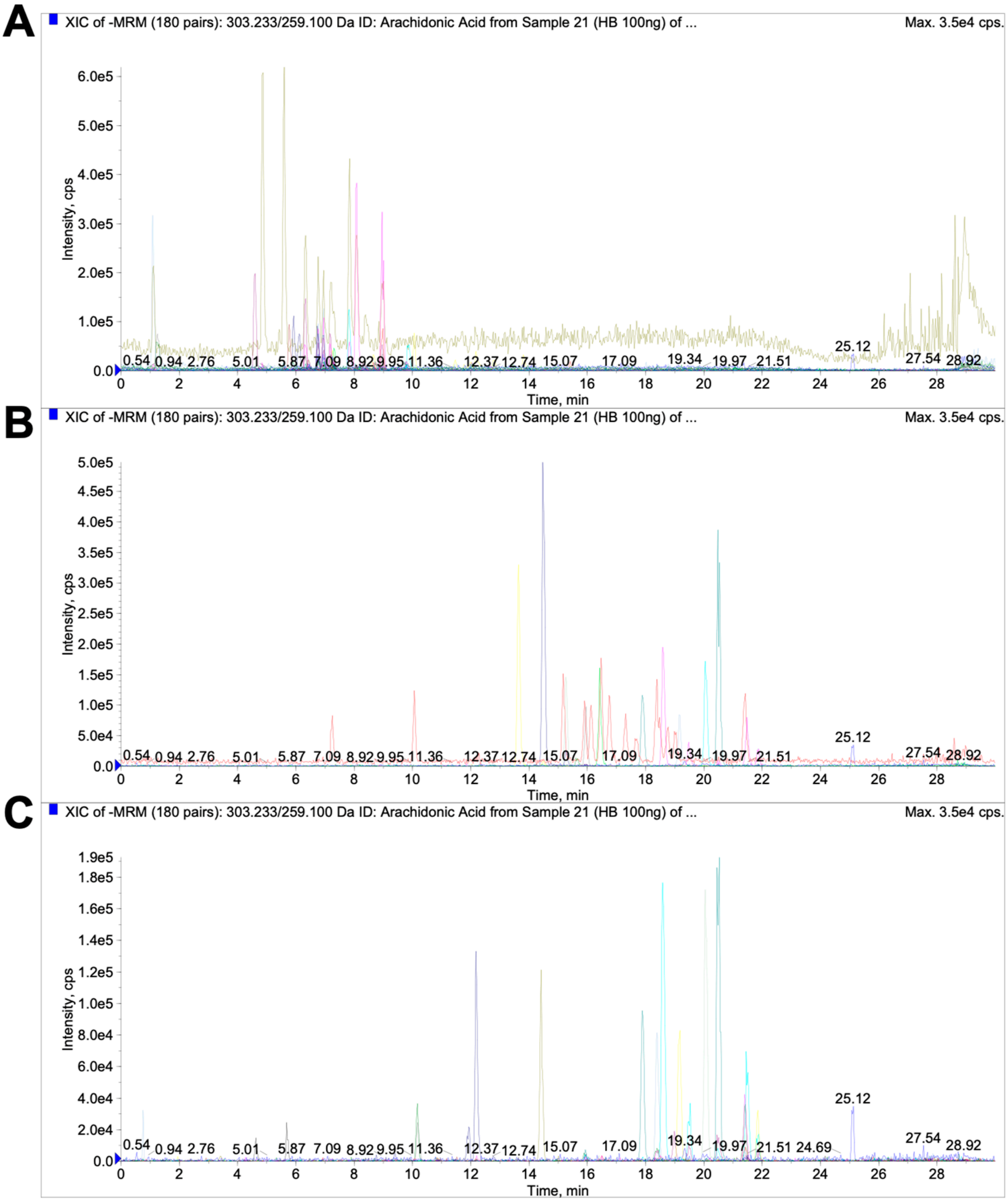

Figure S2. MRM chromatograms of arachidonic acid (AA) and its metabolites derived from the COX, CYP450, and LOX pathways. (A) COX-derived metabolites, comprising 39 analytes; (B) CYP450-derived metabolites, comprising 11 analytes; (C) LOX-derived metabolites, comprising 19 analytes.

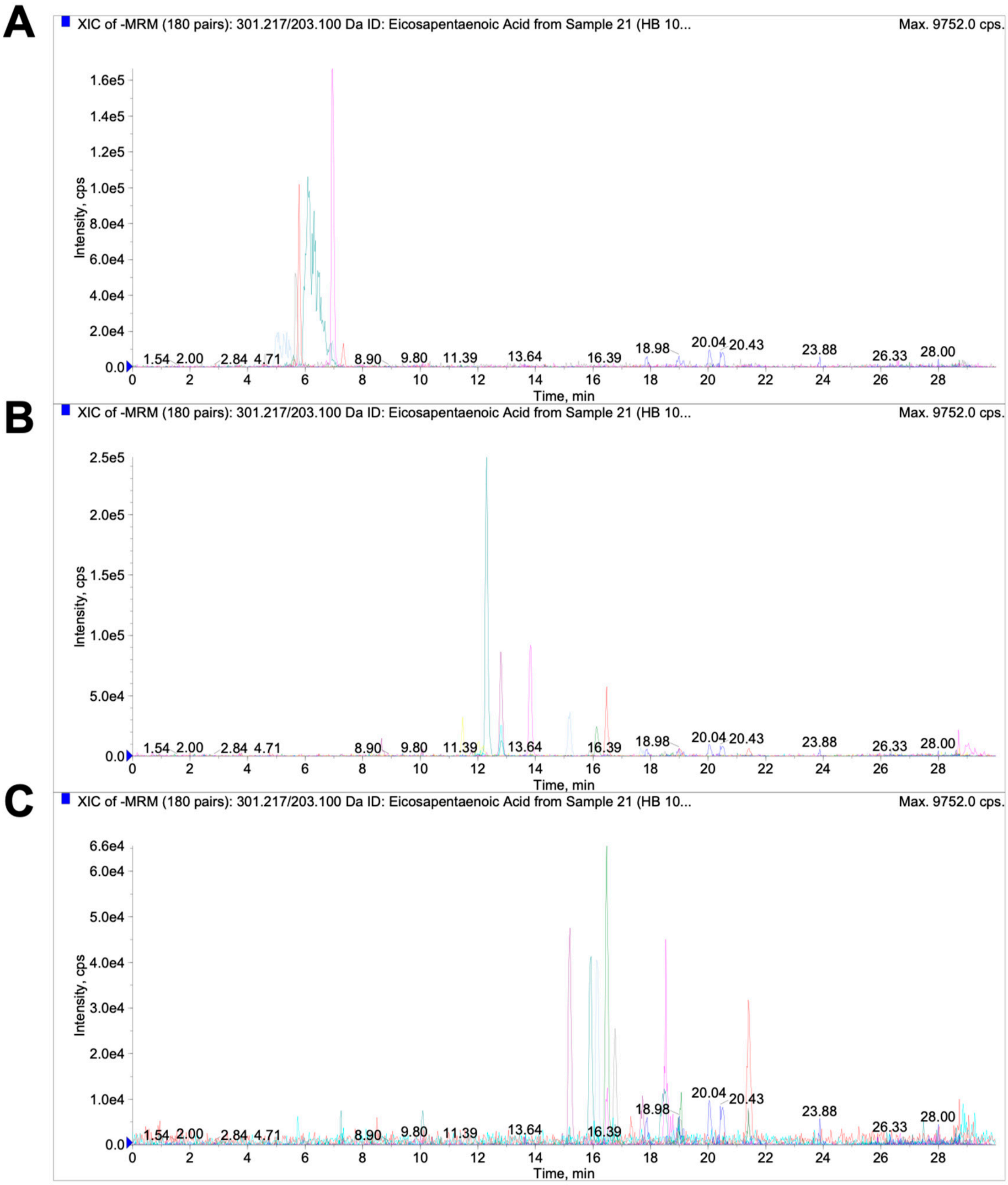

Figure S3. MRM chromatograms of eicosapentaenoic acid (EPA) and its metabolites derived from the COX, CYP450, and LOX pathways. (A) COX-derived metabolites, comprising 6 analytes; (B) CYP450-derived metabolites, comprising 9 analytes; (C) LOX-derived metabolites, comprising 8 analytes.

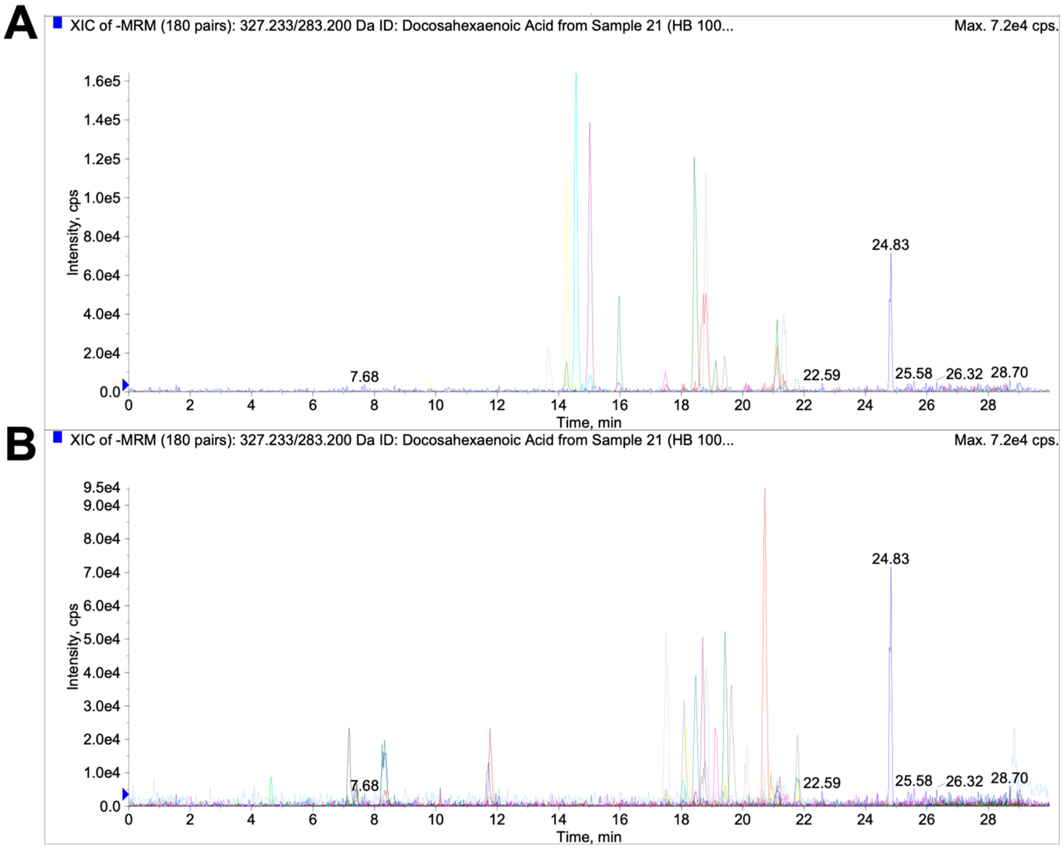

Figure S4. MRM chromatograms of docosahexaenoic acid (DHA) and its metabolites derived from the CYP450 and LOX pathways. (A) CYP450-derived metabolites, comprising 10 analytes; (B) LOX-derived metabolites, comprising 19 analytes.

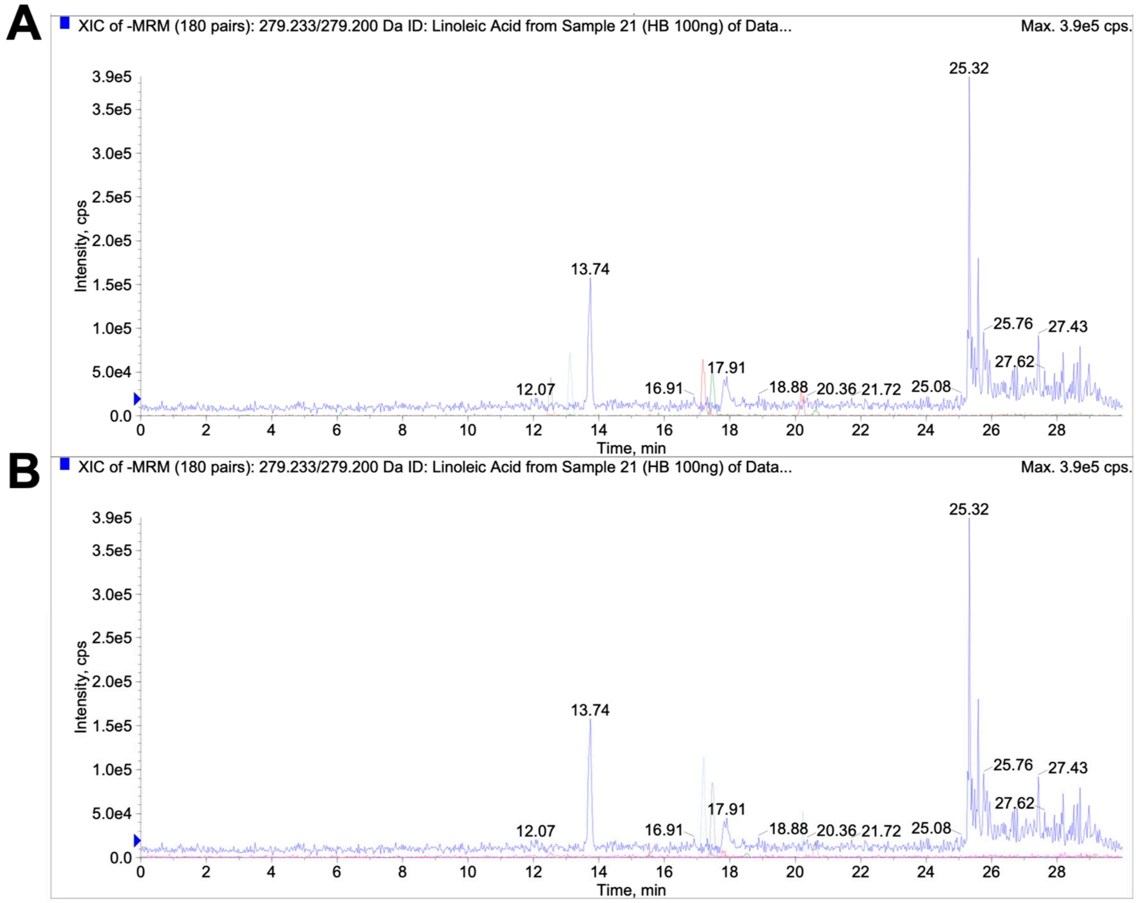

Figure S5. MRM chromatograms of linoleic acid (LA) and its metabolites derived from the CYP450 and LOX pathways. (A) CYP450-derived metabolites, comprising 4 analytes; (B) LOX-derived metabolites, comprising 5 analytes.

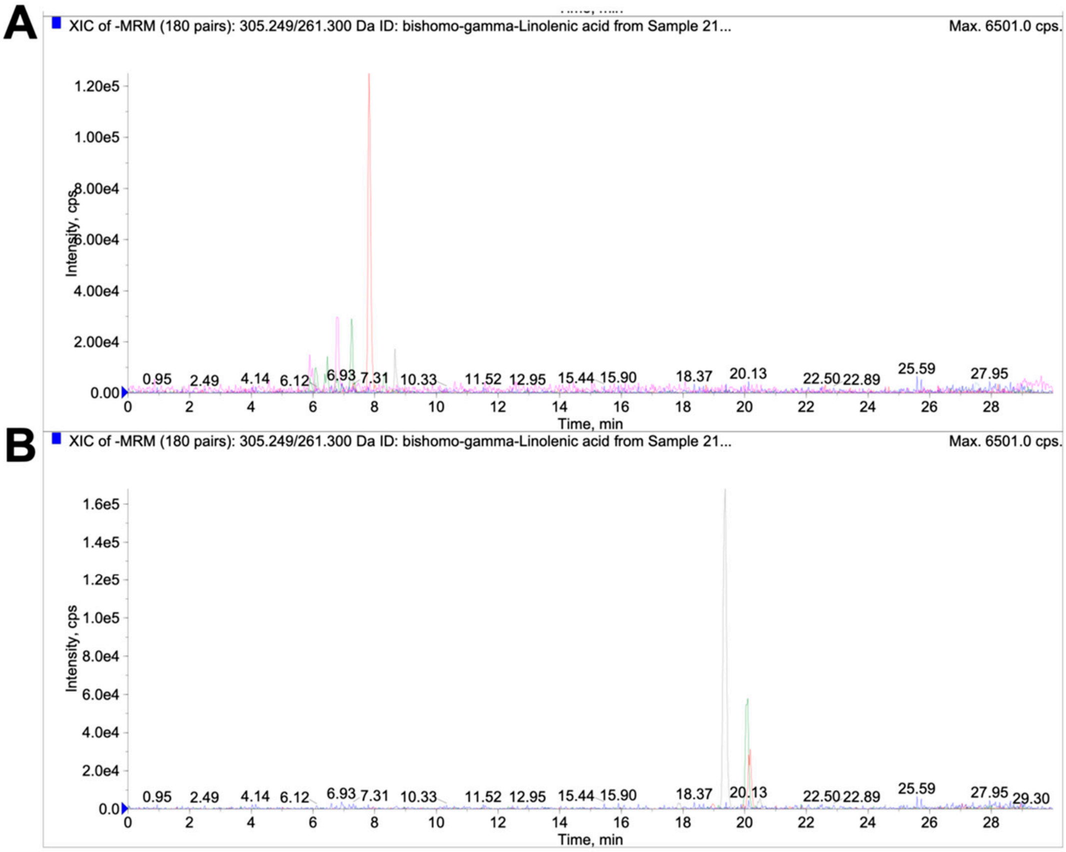

Figure S6. MRM chromatograms of eicosapentaenoic acid (EPA) and its metabolites derived from the COX and LOX pathways. (A) COX-derived metabolites, comprising 5 analytes; (B) LOX-derived metabolites, comprising 3 analytes.

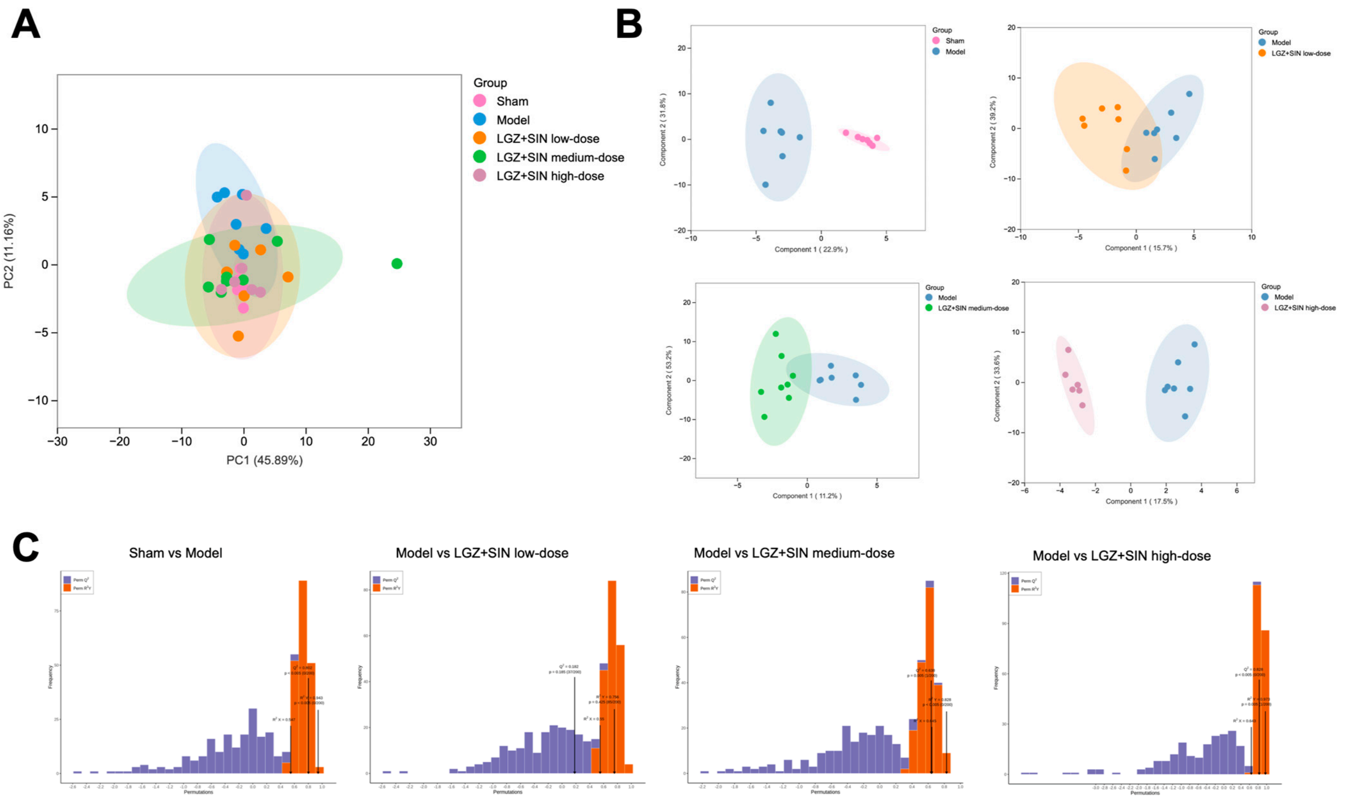

Figure S7. Multivariate statistical analysis of the neurotransmitter metabolic profiles in the spinal dorsal horn of CCI rats following combined ligustrazine and sinomenine treatment. (A) PCA score plot of the experimental groups; (B) OPLS-DA score plots for the indicated group comparisons; (C) permutation test results for the OPLS-DA models.

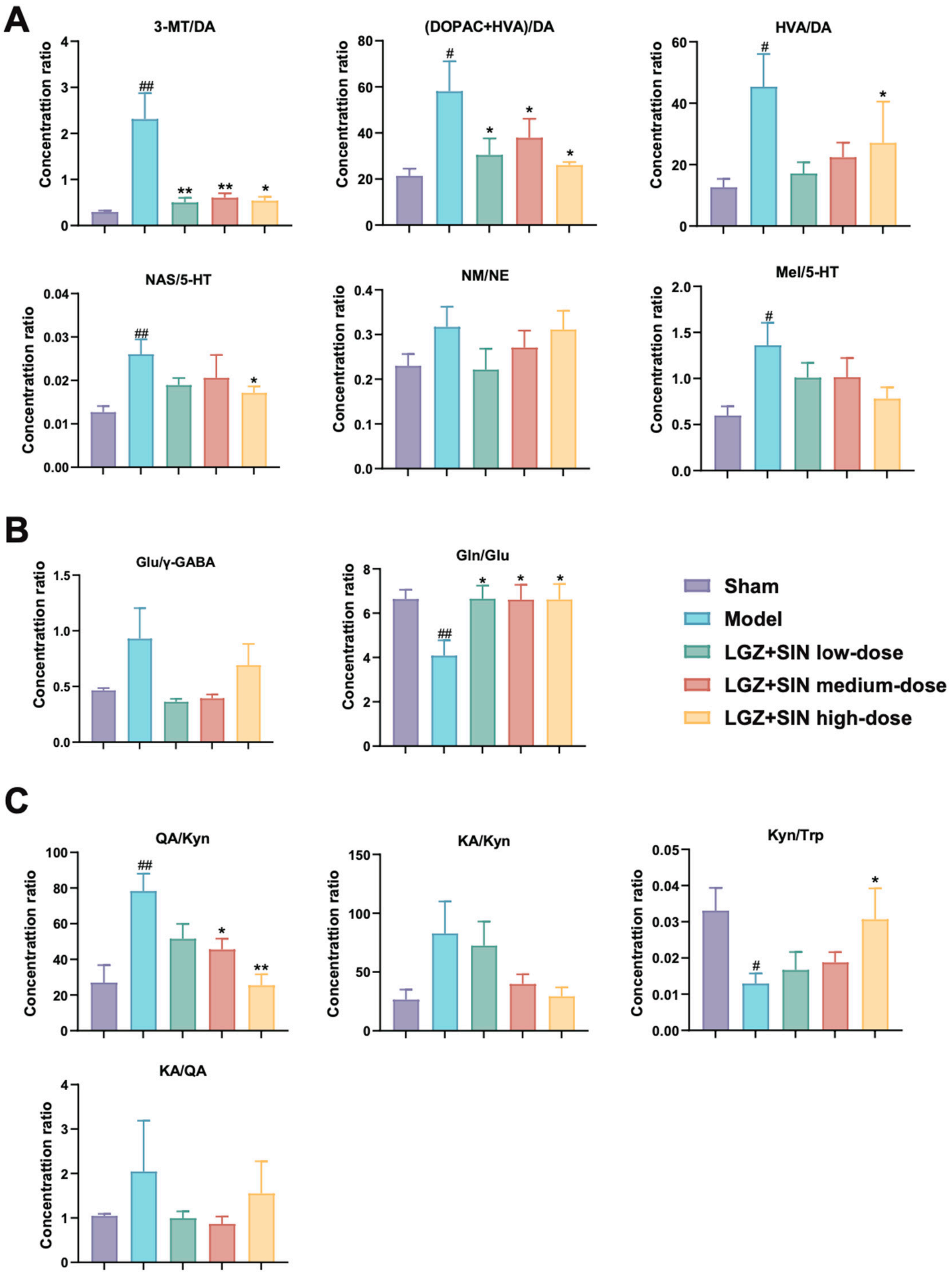

Figure S8. Effects of combined ligustrazine and sinomenine treatment on neurotransmitter metabolic turnover and homeostatic balance in the spinal dorsal horn of CCI rats. (A) Indices of monoamine neurotransmitter turnover; (B) indices related to excitatory/inhibitory balance; (C) indices of metabolic homeostasis within the kynurenine pathway. All ratio-based indices are expressed as metabolite peak-area ratios. Data are presented as the mean ± SEM. $^{\#}P < 0.05$ and $^{\#\#}P < 0.01$ vs. the Sham group; $^{*}P < 0.05$ and $^{**}P < 0.01$ vs. the Model group.

Supplementary Table S1-S3

Table S1. qRT-PCR primer sequences

Gene	Primer sequence (5′–3′)
SAT1 (rat)	FORWARD: ATCCTGCGACTGATCAAGGAAC
	REVERSE: TCAGGGGTCCAGTGCTCTTT
SLC7A11/xCT (rat)	FORWARD: AACTGCTGGTAATACGCCCC
	REVERSE: GGAAAATCTGGATCCGGGCA
GRIN2B (rat)	FORWARD: CAGCTGTTTGGAGATGGGGA
	REVERSE: GAAGGTGATGAGGCTGAGGG

Table S2. Overlapping differential metabolites identified from Model versus Sham and LGZ + SIN versus Model comparisons

Metabolite	KEGG ID	HMDB ID	Model vs Sham			LGZ+SIN vs Model		
			VIP	Fold Change	P value	VIP	Fold Change	P value
3-(4-Hydroxyphenyl) Pyruvate	C01179	HMDB0000707	2.042624244	2.594712937	0.002175602	1.455521458	0.38551326	0.008158508
3-Hydroxy-3-Methylglutaryl-CoA	C00356	HMDB0001375	2.710287799	2.447531585	0.000621601	2.310293279	0.206186778	0.000106459
NADPH	C00005	HMDB0000221	2.055793983	2.318443641	0.009324009	1.779331657	0.20335182	0.001165501
Deoxycytidine Monophosphate	C00239	HMDB01202	2.167636686	2.658175794	0.013986014	1.489754661	1.763107351	0.034965035
Adenosine Triphosphate	C00002	HMDB0000538	2.58463479	0.447573966	0.000434631	1.729553065	0.308596324	0.002331002
Cholesteryl Oleate	C14641	HMDB0000918	1.613118336	0.403684767	0.04009324	2.193216508	0.174506781	0.001165501

Table S3. Overlapping expressed genes identified from Model versus Sham and LGZ + SIN versus Model comparisons

Gene ID	Gene name	Model vs Sham		LGZ+SIN vs Model	
		Fold Change	P value	Fold Change	P value
ENSRNOG00000001130	Nos1	0.686619433	0.006988841	1.444408752	0.013218493
ENSRNOG00000002918	FAM187A	1.744753966	0.002263226	0.667992275	0.017257023
ENSRNOG00000003136	Fcrla	3.356758466	0.002403164	0.291738041	0.001752868
ENSRNOG00000006885	Dbx2	1.405879356	0.020510765	0.70376705	0.015481894
ENSRNOG00000007081	Xdh	0.717101632	0.006454872	1.393481223	0.035851796
ENSRNOG00000012151	Itpripl1	1.443847035	0.021581108	0.630161277	0.002691483
ENSRNOG00000012660	Postn	0.52677745	0.044059344	2.259701222	0.01681234
ENSRNOG00000012980	Hyls1	0.686518651	0.046892093	1.472871805	0.017542779
ENSRNOG00000014666	Tfeb	1.268415588	0.036104199	0.812384104	0.041828448
ENSRNOG00000018691	Ccnl2	0.770091796	0.003335062	1.213488246	0.021311016
ENSRNOG00000019387	Ifi30	1.577849333	2.18029E-05	0.804819801	0.028401845
ENSRNOG00000021015	Sbsn	0.468488841	0.02295131	2.027228735	0.039565782
ENSRNOG00000024899	Cxcl13	16.64212459	2.64371E-18	0.565093148	0.034257681
ENSRNOG00000026604	Cercam	0.603951189	0.004686505	1.430511446	0.036245729
ENSRNOG00000046790	AABR07040887.1	0.438212313	0.000984575	2.00561216	0.013938356
ENSRNOG00000048356	Pum3l1	0.399132146	0.000544813	2.310410736	0.002856152
ENSRNOG00000050067	LOC120097492	1.463927525	0.002072976	0.766284664	0.025659637
ENSRNOG00000055608	Zfp94l1	1.241491214	0.01787394	0.816018987	0.015440549
ENSRNOG00000062707	LOC120100725	0.679804395	0.001022642	1.383686482	0.012347808
ENSRNOG00000072828	-	0.627987092	0.000587045	1.393957187	0.022680742
ENSRNOG00000073706	-	277.1019701	1.90387E-10	0.006228483	9.69491E-09
ENSRNOG00000073861	-	14.50168196	0.003181745	0.251134777	0.033675487
ENSRNOG00000077115	Tmed8	0.775881609	0.042237128	1.282256186	0.046929953
ENSRNOG00000079000	-	0.151419312	0.036110054	7.103707611	0.022640099
ENSRNOG00000081786	-	0.58221851	0.04848801	1.718157702	0.046458849
ENSRNOG00000081922	-	0.49008532	0.002185375	1.755564988	0.015382352
ENSRNOG00000082453	-	0.338701734	0.047247976	3.221914218	0.025437358
ENSRNOG00000084910	-	411.2296259	2.47606E-08	0.002155177	1.28769E-08
ENSRNOG00000087542	-	0.766244393	0.024509387	1.488985568	8.97048E-05
ENSRNOG00000088952	-	0.54687933	0.032627749	1.90692978	0.042925056
ENSRNOG00000089373	-	1.554262603	0.000756008	0.668104462	0.000244006
ENSRNOG00000089834	-	0.576606317	0.015284057	1.636881844	0.016962507
ENSRNOG00000090806	-	0.402472878	0.000163539	2.504311998	0.001727634
ENSRNOG00000091132	-	0.092781217	0.003271189	8.095885733	0.018291631
